# miR-379 deletion ameliorates features of diabetic kidney disease by enhancing adaptive mitophagy via FIS1

**DOI:** 10.1038/s42003-020-01516-w

**Published:** 2021-01-04

**Authors:** Mitsuo Kato, Maryam Abdollahi, Ragadeepthi Tunduguru, Walter Tsark, Zhuo Chen, Xiwei Wu, Jinhui Wang, Zhen Bouman Chen, Feng-Mao Lin, Linda Lanting, Mei Wang, Janice Huss, Patrick T Fueger, David Chan, Rama Natarajan

**Affiliations:** 1 Department of Diabetes Complications and Metabolism, Diabetes and Metabolism Research Institute, Caltech, 1200 East California Boulevard, Pasadena, CA 91125 USA; 2 Transgenic Mouse Facility, Center for Comparative Medicine, Caltech, 1200 East California Boulevard, Pasadena, CA 91125 USA; 3 Integrative Genomics Core, Caltech, 1200 East California Boulevard, Pasadena, CA 91125 USA; 4 Irell and Manella Graduate School of Biological Sciences, Caltech, 1200 East California Boulevard, Pasadena, CA 91125 USA; 5 Department of Cellular and Molecular Endocrinology, Caltech, 1200 East California Boulevard, Pasadena, CA91125 USA; 6 Comprehensive Metabolic Phenotyping Core, Beckman Research Institute of City of Hope, 1500 East Duarte Road, Duarte, CA 91010 USA; 7 Division of Biology and Biological Engineering, Caltech, 1200 East California Boulevard, Pasadena, CA 91125 USA

**Keywords:** End-stage renal disease, Genetic engineering

## Abstract

Diabetic kidney disease (DKD) is a major complication of diabetes. Expression of members of the microRNA (miRNA) miR-379 cluster is increased in DKD. miR-379, the most upstream 5′-miRNA in the cluster, functions in endoplasmic reticulum (ER) stress by targeting EDEM3. However, the in vivo functions of miR-379 remain unclear. We created miR-379 knockout (KO) mice using CRISPR-Cas9 nickase and dual guide RNA technique and characterized their phenotype in diabetes. We screened for miR-379 targets in renal mesangial cells from WT vs. miR-379KO mice using AGO2-immunopreciptation and CLASH (cross-linking, ligation, sequencing hybrids) and identified the redox protein thioredoxin and mitochondrial fission-1 protein. miR-379KO mice were protected from features of DKD as well as body weight loss associated with mitochondrial dysfunction, ER- and oxidative stress. These results reveal a role for miR-379 in DKD and metabolic processes via reducing adaptive mitophagy. Strategies targeting miR-379 could offer therapeutic options for DKD.

## Abstract (web summary)

Kato, Abdollahi et al. identify a redox protein thioredoxin and mitochondrial fission-1 (FIS1) protein as miR-379 targets in mouse kidney. They find that miR-379 knockout mice are protected from diabetic kidney disease by enhancing mitophagy via FIS1, suggesting miR-379 as a potential therapeutic target for diabetic kidney disease.

## Introduction

Both type 1 and type 2 diabetes (T1D and T2D) are associated with significantly accelerated complications, including renal disease, known as diabetic kidney disease (DKD)^1–5^. To date, there are very few effective drugs for DKD and affected individuals often succumb to end-stage renal disease needing dialysis or renal replacement, underscoring the urgent need to explore better drug targets. Key features of DKD include renal fibrosis and hypertrophy due to accumulation of extracellular matrix (ECM) proteins such as collagens and fibronectin in renal glomerular and tubular compartments, as well as albuminuria and podocyte dysfunction^1–5^. Metabolic alterations, including mitochondrial dysfunction and oxidative stress, in most renal cells, including tubular epithelial, glomerular mesangial and podocytes, are associated with progressive DKD^3,5,6^.

Non-coding RNAs such as microRNAs (miRNAs) and long-non-coding RNAs (lncRNAs) regulate gene expression via posttranscriptional and epigenetic mechanisms. miRNAs are short non-coding RNAs that target about 60% of the genome and alter gene expression by base-pairing with complementary “seed” sequences in the 3′-untranslated region (3′-UTR) of their target genes^7^. lncRNAs are longer transcripts such as messenger RNAs (mRNAs) but lack protein-coding (translation) potential^8,9^. miRNAs and lncRNAs regulate cellular functions and pathophysiological conditions associated with human disease including kidney dysfunction and DKD^3,10^. We and others have used in vitro mechanistic and in vivo models to describe the functional roles and actions of several miRNAs, e.g., miR-192, miR-21, miR-93, miR-200, miR-29, as well as various lncRNAs in the pathogenesis of DKD, and also evaluated the translation potential of targeting them as treatment options for DKD^3,10–12^.

We recently established that a megacluster of miRNAs (including miR-379 and others), along with its host lncRNA (lncMGC), is upregulated via endoplasmic reticulum (ER) stress in the kidneys of diabetic mice and induces early features of DKD such as hypertrophy and fibrosis^13^. To inhibit lncMGC, we designed a GapmeR (locked nucleic acid and phosphorothioate-modified antisense oligonucleotide), which efficiently inhibited expression of the miR-379 cluster and lncMGC, and attenuated features of early DKD in mice^13^. We also showed that miR-379, the first miRNA (5′-end) in the megacluster, targets and downregulates the ER degradation enhancer, mannosidase α-like 3 (EDEM3)^13,14^, in glomerular mesangial cells, suggesting it may have metabolic functions in the kidney.

We designed the present study to delineate the in vivo functional role of miR-379 in the kidney under diabetic conditions and the putative DKD-related functions of its targets. Transgenic and genetic knockout (KO) mutant mice provide useful models for studying diabetes and diabetic complications. Generation of mutant mice has become much faster and more efficient with the CRISPR-Cas9 editing method. We therefore created miR-379KO mice using the CRISPR-Cas9 nickase technique^15^. Although miRNA targets are typically predicted by matching seed sequences and flanking sequences using in silico techniques^7,16,17^, additional targets have recently been identified more reliably by experimental methods that examine direct interaction of miRNAs and target RNAs in living cells using a strategy that combines immunoprecipitation (IP) of components of the RNA-induced silencing complex [RISC; e.g., argonaute (AGO) proteins], ligation of AGO-associated RNA–RNA duplexes to form chimeric RNAs, and subsequent RNA sequencing (RNA-seq)^18–20^. As analysis of data from such CLASH (cross-linking, ligation, and sequencing of hybrids) method does not rely on bioinformatics prediction algorithms, the miRNA targets identified in this unbiased manner from AGO-IP can be further evaluated as therapeutic targets for various human diseases, including DKD. In this study, we identified new miR-379 targets related to oxidative stress, ER stress, and mitochondrial dysfunction using the unbiased AGO2-CLASH technique^19^ by comparing mouse glomerular mesangial cells (MMCs) isolated from wild-type (WT) and miR-379KO mice. We validated the expression of these new targets in vivo using WT and miR-379KO mice. MMC from miR-379KO mice depicted protection from high-glucose (HG)-induced metabolic and mitochondrial dysfunction through enhanced adaptive mitophagy. Importantly, we showed that, in a streptozotocin (STZ)-injected model of T1D, miR-379KO mice were protected from key features of DKD, including renal dysfunction, glomerular expansion, renal fibrosis, and glomerular basement membrane (GBM) thickening, compared to corresponding diabetic WT mice.

## Results

### Generation of miR-379KO mice by CRISPR-Cas9 genome editing

**Fig. 1:**
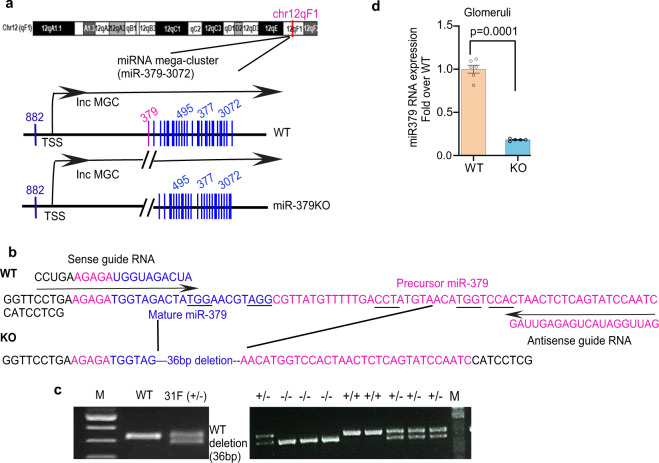
Generation of miR-379 knockout (KO) mice by CRISPR-Cas9 editing. **a** Schematic genomic location of the miR-379 megacluster (MGC) of miRNAs (miR-379-3072) and structure of wild-type (WT) and miR-379 knockout (KO) mice. The miR-379 MGC is located on mouse chromosome 12 within the host lncRNA (lncMGC) and consists of ~40 miRNAs. miR-379 is the most 5′-miRNA, miR-495, miR-377, and miR-3072 are in the middle, and miR-882 is located far upstream of cluster. **b** Sequences of miR-379 genomic regions WT (upper) and KO (lower, 36 bp deletion). Positions of guide RNAs used for editing by CRISPR-Cas9 approach are shown by arrows. Sequences of mature miR-379 are represented in blue and the precursor miR-379 in pink. PAM sequences (NGG) are underlined. **c** Left, genotypes of WT and a founder (31F) mouse from tail DNA samples. The founder [31F (+/−), left panel] shows a 36 bp shorter PCR fragment (i.e., deletion of miR-379). Lane M: molecular weight markers. Right, germline transmission of miR-379 deletion. The female founder heterozygote (31F^+/−^) was crossed with a WT male and the deletion was transmitted to the next generation; multiple heterozygotes (+/−), WT, and/or miR-379 deletion are shown. Homozygotes (−/−, miR-379 deletion) were obtained by crossing heterozygous mice. The miR-379KO mouse colony was expanded by crossing the homozygous mice. **d** Significant decrease of glomerular miR-379 RNA expression in miR-379KO mice compared to WT mice (*n* = 5–6/group). Statistical analyses for two groups were performed by Student’s *t*-test. Data are presented as mean ± SEM.

Figure 1a shows the schematic genome structure of the mouse lncMGC region which hosts the miR-379 megacluster, in which miR-379 is the most 5′-miRNA within the cluster and miR-882 is outside. For CRISPR-Cas9-mediated generation of miR-379KO in mice, we designed guide RNAs as described in the “Methods.” The guide RNAs (Fig. 1b) were first verified for cleavage activity in vitro using the TCMK1 mouse kidney cell line (see “Methods”). We then created miR-379KO mice by injecting fertilized eggs from C57BL/6J mice with guide RNAs and RNA encoding CRISPR-Cas9 nickase. We sequenced the PCR fragments from mouse genomic DNA samples to confirm that several surviving mice showed the 36 bp deletion of the miR-379 region (Fig. 1c). We crossed the third generation of these founder mice with WT C57BL/6J mice and obtained homozygous miR-379KO mice (homozygotes, −/−) by intercrossing germline-transmitted heterozygous mice (heterozygotes, +/−) (Fig. 1c). We confirmed significant reduction of miR-379 in glomeruli isolated from kidneys of miR-379KO mice compared to WT mice (Fig. 1d, *P* < 0.0001). The miR-379KO mice appeared normal and did not depict any overt defects or abnormalities.

### AGO2-IP-CLASH screening identifies novel targets of miR-379

**Fig. 2:**
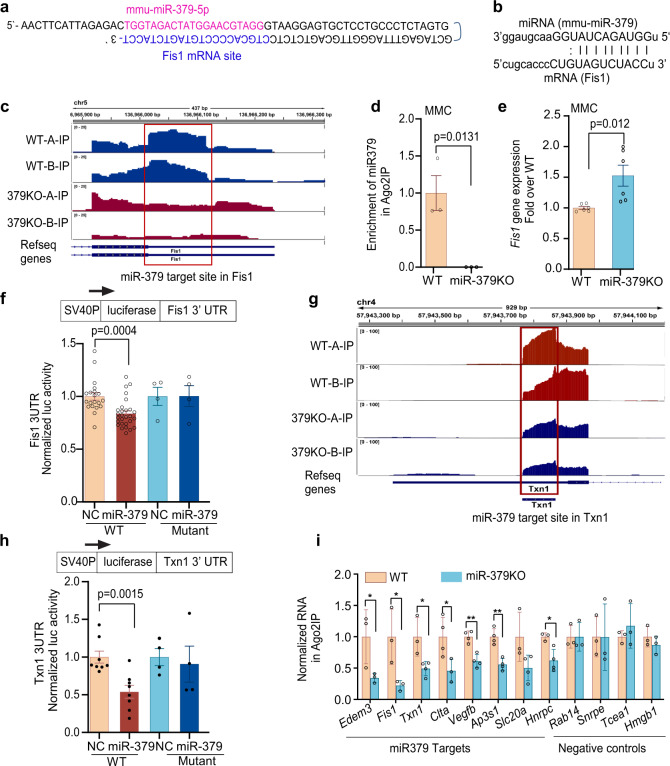
Identification of miR-379 targets. **a** Example of hybrid sequence of miR-379 in pink and Fis1 in blue. **b** Alignment of miR-379 and its target site in the Fis1 3′-UTR (miRanda; microRNA.org). **c** Enrichment of AGO2 IP-seq RNA reads at miR-379 target site in Fis1 3′-UTR in WT mouse mesangial cells (MMC), with notable reduction in miR-379KO MMC. Two independent samples (A and B) from WT MMC (WT-A-IP and WT-B-IP) and miR-379KO MMC (379KO-A-IP and, 379KO-B-IP) were examined. **d** Enrichment of miR-379 RNA in AGO-IP in MMC isolated from kidney glomeruli of WT mice and significant reduction in miR-379KO MMC (*n* = 3/group). **e** Significant increase of *Fis1* gene expression in miR-379KO MMC compared to WT MMC, suggesting Fis1 is a target of miR-379 (*n* = 6/group). **f** Significant decrease of WT Fis1 3′-UTR luciferase reporter activity by transfection with miR-379 mimic oligonucleotide, compared to mutant Fis1 3′-UTR reporter under similar conditions, further supporting Fis1 3′-UTR to a true target of miR-379. **g** Enrichment of AGO2 IP-seq RNA reads at the 3′-UTR of *Txn1* gene in WT MMC and its significant reduction in miR-379KO MMC. Two independent samples (A and B) from WT MMC (WT-A-IP and WT-B-IP) and miR-379KO MMC (379KO-A-IP and 379KO-B-IP) were examined. **h** Significant decrease of WT *Txn1* 3′-UTR luciferase reporter activity induced by miR-379 mimics, compared with no change in mutant *Txn1* 3′-UTR reporter by miR-379 mimics. NC, negative control mimic; miR-379, miR-379 mimic. **i** RT-qPCR validation of the expression of enriched candidate genes identified by AGO2 IP-seq. RNA expression of all eight candidate miR-379 targets tested was decreased in AGO2-IP from miR-379KO MMC compared to WT MMC. *Rab14*, *Snrpe*, *Tcea1*, and *Hmgb1* were used as negative controls because their enrichments in AGO2 IP-seq were not significantly changed between miR-379KO and WT MMC. Each dot indicates one biological repeat. Statistical analyses for two groups were performed by Student’s t-test, and for multiple comparisons one-way ANOVA with Tukey’s post hoc test was used. *P < 0.05, **P < 0.01. All data are presented as mean ± SEM.

The AGO-CLASH technique allows unbiased high-throughput identification of miRNA targets by ligation and sequencing of miRNA-target RNA duplexes associated with AGO2 protein, a key component of the RISC complex. We performed AGO2-CLASH as described^19,20^ (see “Methods” and Supplementary Fig. 1) using MMC isolated from WT and miR-379KO mice. We also immunoprecipitated the AGO2 RISC complex with anti-mouse AGO2 beads. After phosphorylation of the RNA 5′-ends and ligation using T4 RNA ligase, we extracted the ligated RNAs and subjected them to RNA-seq. To identify bona fide targets of miR-379, we compared RNA-seq data from total RNAs, AGO2-IP RNAs, and AGO2-IP-CLASH RNAs between WT and miR-379KO MMCs (Supplementary Fig. 1). From data analyses, we found several promising candidate targets of miR-379 (i.e., reads highly enriched at their respective 3′-UTRs in WT cells but decreased in miR-379KO cells). One of these was *Fis1*, because we identified sequences at the 3′-UTR of *Fis1*, which hybridized with miR-379 following AGO2-IP in WT but not in miR-379KO MMC (Fig. 2a and Supplementary Table 1). We observed extensive sequence complementarity between miR-379 and the *Fis1* 3′-UTR (Fig. 2b). We also detected significant enrichment of RNA-seq reads at the *Fis1* 3′-UTR following AGO2-IP in WT but not miR-379KO MMC (Fig. 2c). We confirmed the absence of miR-379 following AGO2-IP in miR-379KO MMC using quantitative PCR (qPCR) (Fig. 2d) and, conversely, we observed a significant increase in *Fis1* gene expression in miR-379KO MMC compared to WT MMC (Fig. 2e), suggesting *Fis1* is a bona fide target of miR-379. This was of interest, because FIS1 is a key mitochondrial protein^21,22^. We next performed luciferase reporter assays using a psiCheck2 vector in which we cloned the 3′-UTR of *Fis1*, which demonstrated that *Fis1* 3′-UTR luciferase activity was significantly decreased in MMCs treated with miR-379 mimic oligonucleotides (oligos) compared to negative control (NC) oligos in WT MMC (Fig. 2f). This decrease was not observed in cells transfected with the *Fis1* 3′-UTR vector harboring a mutation in the miR-379 binding site, further confirming *Fis1* to be a target of miR-379 (Fig. 2f).

From comparisons of AGO2-IP RNAs, besides *Fis1 (*Fig. 2c) encoding the mitochondrial fission protein FIS1^22–24^, we also examined other candidate targets of miR-379 with putative functions related to kidney disease. We confirmed *Edem3* as a target, which we previously identified^13^, and also identified interesting new candidate miR-379 target genes, including those encoding the redox protein *Txn1* (Fig. 2g), the growth factor *Vegfb*, the transporter protein *Slc20a1*, the RNA-binding protein *Hnrnpc*, the clathrin protein *Clta*, and the adapter protein *Ap3s1* (Supplementary Fig. 2a–e and Supplementary Table 2a, b).

Interestingly, Ingenuity pathway analyses (IPAs) of the RNA-seq data revealed pathways related to mitochondrial function (mitochondrial depolarization, transition, and permeability of mitochondria), as well as renal injury (renal ischemia, acute renal failure, glomerular injury, hypertrophy) were enriched in the differentially expressed genes between WT and miR-379KO MMC (Supplementary Fig. 3a, b). These data suggest that the targets of miR-379 are involved in processes related to mitochondrial and renal function.

We further validated *Txn1*, because it encodes a redox protein, thioredoxin, and we also detected significant enrichment of RNA-seq reads at the *Txn1* 3′-UTR following AGO2-IP in WT but not miR-379KO MMC (Fig. 2g). We again performed luciferase reporter assays using a psiCheck2 vector harboring the *Txn1* 3′-UTR, which showed that *Txn1* 3′-UTR luciferase activity was significantly decreased by miR-379 mimic oligos compared to NC in WT MMC. In contrast, we detected no change in cells transfected with a miR-379-binding site mutant *Txn1* 3′-UTR reporter luciferase vector, further confirming that *Txn1* is a real target of miR-379 (Fig. 2h). We also further validated the RNA-seq data from AGO2-IP RNAs by performing qPCR validations, which confirmed the significant decrease in enrichments of *Edem3*, *Fis1*, *Txn1*, *Clta*, *Vegfβ*, *Ap3s1*, and *Hnrnpc*, but not *Slc20a1* or NCs, in AGO2-IP from miR-379KO MMC compared to WT (Fig. 2i), verifying they are valid targets of miR-379. Among these, EDEM3 has a key role in regulating ER stress^14^, FIS1 functions in mitochondrial fission and quality control, including mitophagy^22,25^, and TXN1 has several roles, including antioxidant functions, all factors related to DKD^26^. Therefore, increased ER stress, mitochondrial dysfunction, and oxidative stress resulting from miR-379-mediated inhibition of its target genes *Edem3*, *Fis1*, and *Txn1*, respectively, may significantly contribute to DKD.

### miR-379KO MMC are protected from HG-induced mitochondrial dysfunction

**Fig. 3:**
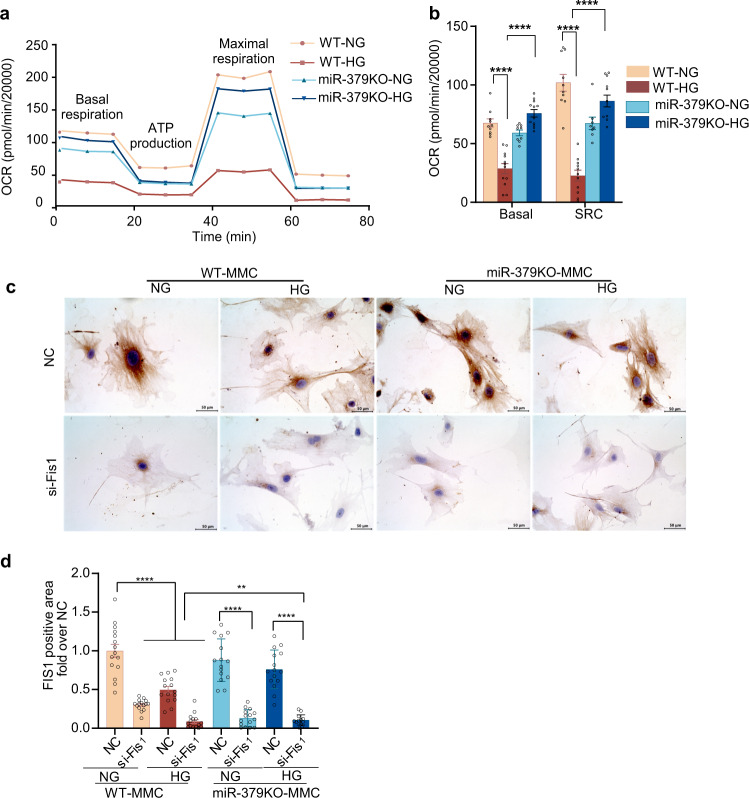
Mitochondrial function assays in MMC under normal and high-glucose conditions. **a** Seahorse XF Cell Mito Stress test for mitochondrial function at basal conditions, ATP production, and maximal respiration levels using MMC from WT or miR-379KO mice cultured with normal glucose (NG, 5.5 mM) or high glucose (HG, 25 mM) for 72 h. **b** Oxygen consumption rates (OCRs) were calculated in basal and spare respiratory capacity (SRC) levels in NG and HG condition (20,000 cells per well of 96-well assay plate). **c** Representative images of IHC staining to detect FIS1 expression (brown color) in negative control (NC) and Fis1 siRNA (si-Fis1)-transfected MMC from WT or miR-379KO mice cultured with NG or HG. **d** Bar graph quantifications showing significant reduction in FIS1 levels in si-Fis1-transfected NG- or HG-treated WT and miR-379KO MMC compared to NC, and significant decrease in FIS1 levels in HG-treated WT MMC but not miR-379KO MMC (*n* = 15 cells/group). The in vitro experiments were performed with at least three biological replicates. One-way ANOVA with Tukey’s post hoc test for multiple comparisons. **P < 0.01, ****P < 0.0001. All data are presented as mean ± SEM. NC, negative control siRNA. si-Fis1, Fis1 siRNA.

As we identified the mitochondrial protein FIS1 as a miR-379 target, we compared mitochondrial function between WT and miR-379KO MMC using a Seahorse XF Cell Mito Stress test. In WT MMC treated with HG (25 mM), we observed a significant decrease in oxygen consumption rate (OCR) at basal respiration, ATP production rate, and maximal respiration compared to WT MMC treated with normal glucose (NG; 5.5 mM) (Fig. 3a, b). Interestingly, miR-379KO MMCs were significantly protected from this HG-induced decrease of mitochondrial function.

As we previously showed miR-379 is upregulated by HG in MMC^13^, we next tested whether HG treatment reduces levels of the miR-379 target, namely the mitochondrial protein FIS1 and whether a loss of FIS1 (by HG or Fis1 siRNA treatment) affects mitochondrial quality and health. We transfected WT MMC with siRNA-targeting *Fis1* (si-Fis1) and verified reduction in FIS1 protein levels by immunohistochemical (IHC) staining relative to NC siRNA (Fig. 3c). Significant reduction of FIS1 by the *Fis1* siRNA was observed in both WT and miR-379KO MMC (Fig. 3c, d, si-Fis1). We found that FIS1 expression was also significantly decreased in WT MMC treated with HG (Fig. 3c, d, WT MMC), further supporting *Fis1* as a target of miR-379 and this is in line with our earlier data showing miR-379 is upregulated by HG^13^. On the other hand, no significant decrease of FIS1 was detected in miR-379KO MMC treated with HG (miR-379KO HG-NC), as compared to WT MMC HG-NC vs. the respective NG-treated cells (Fig. 3c, d). These results are consistent with the Seahorse results showing that mitochondrial activity is maintained at higher levels in miR-379KO MMC even under HG conditions (Fig. 3a, b).

**Fig. 4:**
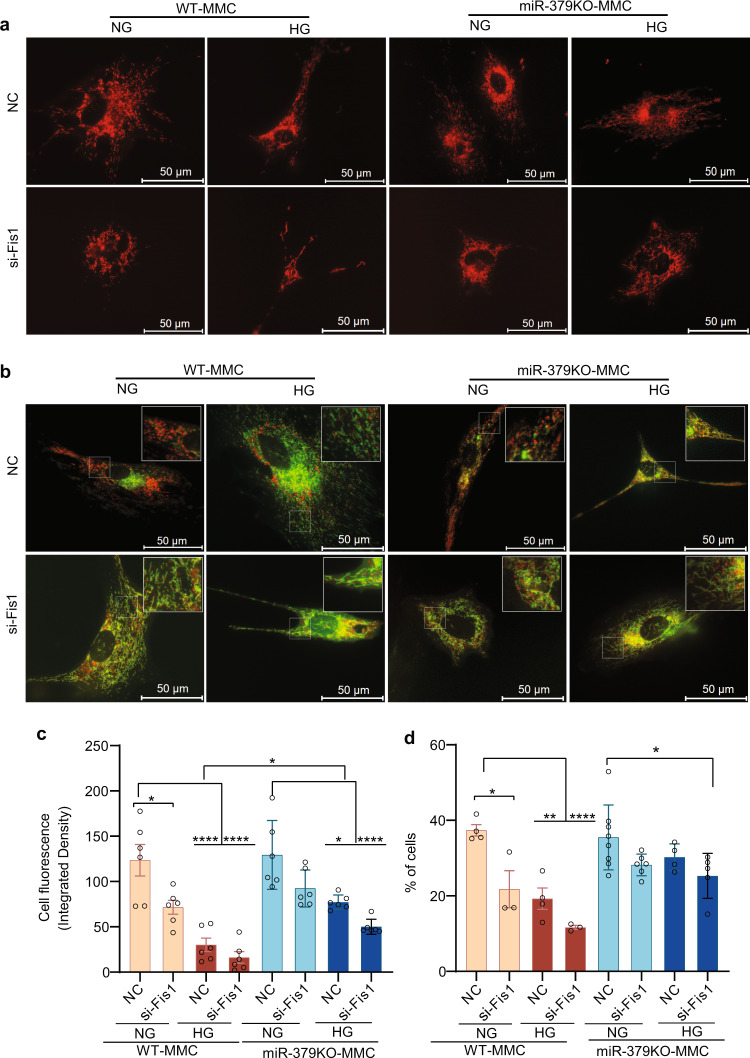
Mitochondrial dysfunction and mitophagy assays in MMC under normal and high-glucose conditions. **a** Representative images of WT and miR-379KO MMC transfected with the DsRed2-Mito-7 plasmid, which fluorescently labels mitochondria with red emission spectra (quantification in **c**). WT and miR-379KO MMC treated with NC siRNA or Fis1 siRNA were transfected with DsRed2-Mito-7 reporter. Upper panel (with NC siRNA): in HG conditions (25 mM glucose), mitochondrial signal intensity (red fluorescence) was significantly reduced compared to NG (5.5 mM glucose) conditions in both WT and miR-379KO MMC, but to a lesser extent in miR-379KO MMC. Lower panel: with si-Fis1: WT-MMC cells treated with si-Fis1 show decreased intensity in mitochondrial fluorescent signals in NG and HG conditions. miR-379KO MMC with si-Fis1 under HG conditions depicted more significant changes than under NG conditions. The degree of reduction of fluorescence (mitochondrial quality) under HG conditions was much lower in miR-379KO MMC compared to WT MMC. **b** Representative images showing adaptive mitophagy in MMC examined by expressing the pCLBW-cox8-EGFP-mCherry reporter (quantification in **d**). WT and miR-379KO MMC MMC treated with NC siRNA or Fis1 siRNA were transfected with pCLBW-cox8-EGFP-mCherry and then treated with HG (25 mM) or NG (5.5 mM) for 5 days at 37 °C and 5% CO_2_. Upper panels (with NC siRNA): adaptive mitophagy shows marked decrease in WT-MMC after 5 days of HG treatment (decrease in red mCherry fluorescence) but not in NG; lower panels (with si-Fis1): Fis1 siRNA significantly reduced mitophagy in WT MMC in NG and HG conditions, but no significant changes were detected in miR-379KO MMC even under HG conditions. Adaptive mitophagy was significantly reduced only in miR-379KO MMC treated with Fis1 siRNA in HG conditions relative to NC under NG conditions. **c** Bar graph quantification of DsRed2-Mito-7 staining data (shown in **a**) based on analysis of integrated density (*n* = 6 cells/group). **d** Bar graph of quantitative analysis of the number of red-only puncta per cell from data in **b** (*n* = 3–8 cells/group). These in vitro experiments in MMC were performed with at least three biological replicates. One-way ANOVA with Tukey’s post hoc tests for multiple comparisons in panels **c** and **d**. *P < 0.05, **P < 0.01, ****P < 0.0001. All data are presented as mean ± SEM. NC, negative control siRNA. si-Fis1, Fis1 siRNA. Scale bar, 50 µm.

To strengthen the results in Fig. 3a, b demonstrating the effects miR-379-mediated loss of FIS1 on mitochondrial function, we transfected WT and miR-379KO MMC with the DsRed2-Mito-7 plasmid, which fluorescently labels mitochondria with red emission spectra (Fig. 4a and quantification in Fig. 4c). The MMC were also transfected with a NC siRNA or si-Fis1. Under HG conditions, mitochondrial signal intensity was significantly reduced compared to NG conditions in both WT-MMC and miR-379KO MMC. However, the degree of reduction of mitochondrial signal intensity was much lesser in miR-379KO MMC (Fig. 4a, NC, upper panel, and Fig. 4c). Moreover, although Fis1 siRNA reduced mitochondrial signal intensity significantly in WT MMC in NG and HG, Fis1 siRNA elicited more significant changes in miR-379KO MMC in HG than in NG (Fig. 4a, si-Fis1 lower panel, and Fig. 4c). These results demonstrate that a combination of HG and si-Fis1 promotes more mitochondrial dysfunction in miR-379KO MMC, unlike in WT MMC, and further support the notion that miR-379-mediated loss of Fis1 promotes mitochondrial dysfunction.

Increasing evidence shows that, although FIS1 is a mitochondrial fission protein, FIS1 can promote adaptive mitophagy to clear damaged mitochondria and is thus important for preserving mitochondrial function and health^23,24,27^. As miR-379 deficiency improved mitochondrial function in MMC and, conversely, siRNA-induced reduction of its mitophagy-related target *Fis1* appeared to worsen mitochondrial function, we next examined whether MMC from miR-379KO mice display better mitochondrial quality and health through adaptive mitophagy under diabetic conditions, compared to WT. Tandem fluorescent-tagged inner mitochondrial membrane protein COX8 (Cox8-GFP-mCherry) reporter monitors mitophagy-based on different pH stability of green fluorescent protein (GFP) and mCherry fluorescent proteins^23,27^. Under steady normal states, cells depict yellow signals in mitochondrial regions, i.e., red (mCherry) plus green (GFP) fluorescence. Under conditions triggering mitophagy, mitochondria are delivered to acidic lysosomes (adaptive mitophagy) where GFP signal is reduced due to its sensitivity to the acidic environment, but mCherry fluorescence remains stable. To monitor the role of Fis1 in adaptive mitophagy (to clear damaged mitochondria), WT and miR-379KO MMC that were again transfected with either a NC siRNA or Fis1 siRNA were transfected with the pCLBW-Cox8-EGFP-mCherry (reporter for mitophagy) and treated with HG (25 mM) or NG (5.5 mM) for 5 days and monitored on day 5. Upper panel in Fig. 4b (NC) and bar graph quantification in Fig. 4d show that, relative to NG conditions, adaptive mitophagy (red fluorescence) was significantly decreased in WT-MMC after 5 days of HG treatment. However, no significant decrease of mitophagy was detected in the miR-379KO MMC treated with HG vs. NG (Fig. 4b, NC, upper panel, and quantification in Fig. 4d). The lower panel of Fig. 4b (si-Fis1) and quantification in Fig. 4d show that, although Fis1 siRNA significantly reduced mitophagy in WT MMC in both NG and HG (vs. corresponding NC siRNA), no significant changes were detected in miR-379KO MMC with si-Fis1 vs. NC siRNA (maintaining high levels of mitophagy). Thus, mitophagy was significantly reduced in WT MMC under HG conditions vs. NG with or without Fis1 siRNA. On the other hand, adaptive mitophagy was reduced only in miR-379KO MMC treated with Fis1 siRNA in HG conditions but not in NG conditions, demonstrating that only a combination of Fis1 siRNA and HG reduces adaptive mitophagy in miR-379KO MMC (Fig. 4b, d). Thus, miR-379KO MMC are more resistant to HG treatment than WT MMC and maintain good quality of mitochondria. Together, these results suggest that adaptive mitophagy is regulated through Fis1 targeted by miR-379 in MMC, and that reduction of adaptive mitophagy due to increased miR-379 in diabetes leads to reduced mitochondrial quality and contributes to mitochondrial dysfunction.

In addition to these parameters of mitophagy and mitochondrial quality/function, we also measured some parameters of autophagy, which is associated with mitophagy. We measured protein expression of ATG5 and P62 (autophagy-related proteins) in mouse kidney glomeruli using IHC staining (Supplementary Fig. 4). ATG5 levels were significantly lower in glomeruli from diabetic WT mice compared to nondiabetic WT mice, whereas no significant changes were observed in miR-379KO mice (diabetic vs. nondiabetic). P62 levels were significantly higher in glomeruli from diabetic WT mice compared to nondiabetic WT mice, but these differences were not observed in diabetic miR-379KO mice vs. corresponding nondiabetic miR-379KO mice.

Taken together, these results indicate that autophagy and related adaptive mitophagy are decreased in MMC from diabetic WT mice relative to diabetic miR-379KO mice. Increased miR-379 under diabetic conditions may promote mitochondrial dysfunction, at least in part by the reduction of its newly identified target *Fis1*.

### miR-379KO mice are protected from diabetes-induced weight loss

**Fig. 5:**
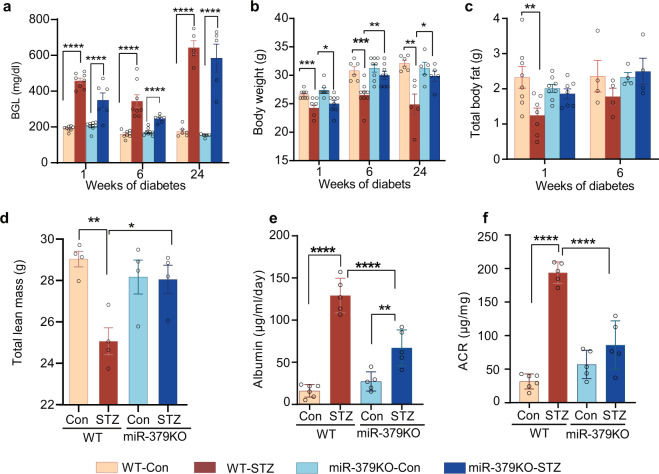
Physiological parameters of diabetic and nondiabetic mice. **a** Diabetes was induced in WT and miR-379KO mice by STZ injections as described in the “Methods.” Non-fasting blood glucose levels (BGL) in WT and miR-379KO mice at indicated time periods during 24 weeks after diabetes onset compared to control (Con) (*n* = 5/group). **b**–**d**, Body weights and body composition analysis using Echo/MRI system. **b** Body weight (*n* = 8/group, *n* = 6–9/group, and *n* = 5–6/group for 1, 6, and 24 weeks post diabetes onset, respectively). **c** Total body fat (*n* = 8 and *n* = 4 for 1 and 6 weeks of diabetes, respectively). **d** Total lean mass (*n* = 4 for 6 weeks of diabetes). **e**, **f** Kidney function (urine albumin excretion) was examined using ELISA in 24 h urine collections after 24 weeks of diabetes. **e** Urine albumin level (*n* = 4–5/group). **f** Albumin/creatinine ratio (ACR) (*n* = 4–5/group). Each dot indicates the value from each mouse. One-way ANOVA with post hoc Tukey’s test for multiple comparisons. *P < 0.05, **P < 0.01, ***P < 0.001, ****P < 0.0001. All data are presented as mean ± SEM.

To next characterize the in vivo role of miR-379 in diabetes, we used STZ injections to induce insulin-dependent diabetes in male WT and miR-379KO mice according to protocols of the NIDDK DiaComp consortium^28^ (Supplementary Fig. 5), and then measured various metabolic parameters of diabetes at 1, 6, and 24 weeks after diabetes onset (Fig. 5). Male mice were used because female mice do not consistently develop diabetes with the multiple low dose STZ injection protocol used^29,30^. Blood glucose levels were monitored and measured at regular intervals (Supplementary Fig. 5b) and mice were killed at 1, 6, or 24 weeks post diabetes. There was no significant difference in the incidence of diabetes between WT and miR-379KO mice. Non-fasting blood glucose levels were significantly higher at individual time points in both WT and miR-379KO diabetic mice compared to their respective nondiabetic controls (Fig. 5a). The average body weight over the period of 24 weeks of diabetes was significantly lower in WT-STZ mice compared to WT nondiabetic controls (Fig. 5b). On the other hand, despite persistent hyperglycemia, body weight loss observed in the WT-STZ mice was significantly ameliorated in miR-379KO-STZ mice, their weights being significantly higher compared to WT-STZ mice at 1, 6, and 24 weeks (Fig. 5b). We performed body composition analysis, which showed that both total body fat and lean mass were significantly reduced over time in WT-STZ mice compared to nondiabetic controls; in contrast, miR-379KO-STZ mice did not display significant reduction in body fat and lean mass (Fig. 5c, d). This protection from diabetes-induced loss of muscle mass in the miR-379KO-STZ mice suggests a key role of miR-379 also in diabetes-induced muscle atrophy.

We next measured metabolic parameters (food, water intake, and movement) and found that they were not significantly different between WT and miR-379KO mice (Supplementary Fig. 6a–c), suggesting that weight loss in WT-STZ mice may not be because of any altered behavior (eating, drinking, or moving). There were also no significant differences in oxygen consumption (VO_2_) or CO_2_ production (VCO_2_) (Supplementary Fig. 6d, e). We measured respiratory exchange rates (RERs) to determine the fuel source in all the four groups of mice, which indicated that fat is the pre-dominant fuel source in both diabetic WT and miR-379KO mice, as they had lower RER compared to their nondiabetic controls (Supplementary Fig. 6f). However, there were no differences between WT and KO mice. Altogether, these data suggest that miR-379 may contribute to metabolic abnormalities, because miR-379KO mice were protected from body weight loss and muscle atrophy in diabetes.

### miR-379KO mice are protected from diabetes-associated albuminuria

As the targets of miR-379 have potential renoprotective properties, we next examined whether miR-379KO mice exhibit renal protection in diabetes. To examine the effects of miR-379 loss on kidney function, we measured albumin and creatinine levels in 24 h urine samples collected from control and STZ-injected diabetic WT and miR-379KO mice at 6 and 24 weeks after diabetes onset. Increased albumin excretion is widely considered to reflect underlying glomerular dysfunction in diabetic mice; measures of the albumin/creatinine ratio (ACR) control for variations in urine flow. At 6 weeks post diabetes induction, there were no differences in albuminuria or creatinine levels among any groups of mice, as expected in this C57BL6 mouse model. However, at 24 weeks post diabetes induction, average urine albumin levels were significantly increased in WT-STZ mice compared to nondiabetic controls and this was significantly attenuated in miR-379KO-STZ mice (Fig. 5e). Consistent with this, at 24 weeks, ACR was ~8-fold higher in WT-STZ mice than in nondiabetic controls and this increase was significantly attenuated in miR-379KO-STZ mice compared to WT-STZ mice (Fig. 5f).

### miR-379KO mice are protected from diabetes-induced glomerular hypertrophy and ECM accumulation

**Fig. 6:**
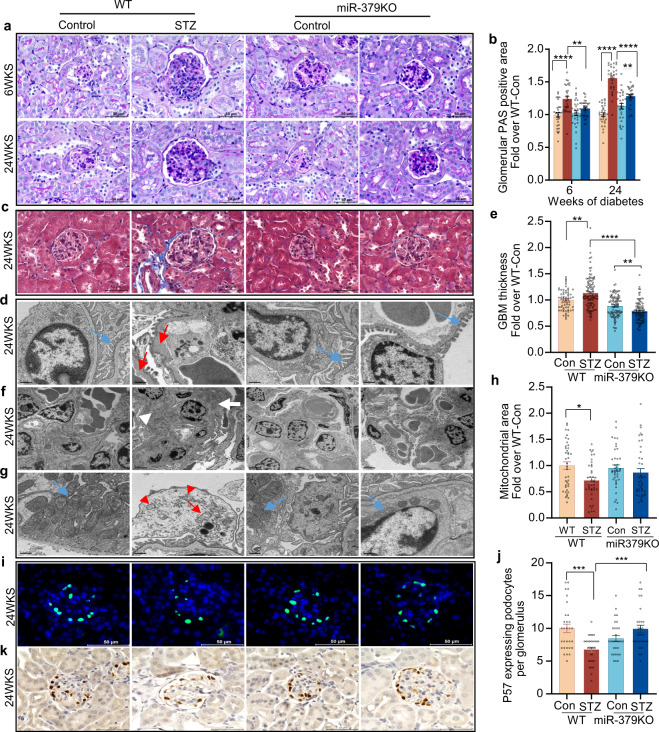
Histological evaluation of kidney cortex samples from WT and miR-379KO mice (control and diabetic). **a** PAS (Periodic acid–Schiff stain) staining in control mice and diabetic mice at 6 and 24 weeks after diabetes onset. Representative images show glomerular mesangial area and extracellular matrix (ECM) accumulation. **b** Quantitative analysis of PAS-positive glomerular areas at 6 and 24 weeks post diabetes induction (*n* = 30 glomeruli/group). **c** Masson’s trichrome staining to detect fibrosis in WT and miR-379KO mice 24 weeks after diabetes onset. Representative images show fibrosis (blue color) in WT-STZ mice that is reduced in miR-379KO mice. Scale bar, 50 µm. **d** Representative images of the glomerular basement membrane (GBM) and podocyte structure using transmission electron microscopy (TEM). Representative TEM images show GBM thickness (Red arrow) and podocyte foot process effacement in WT-STZ mice, whereas these were not observed in miR-379KO-STZ mice (intact podocyte foot processes, blue arrow). Scale bar, 2 µm. **e** Quantitative analysis of GBM (*n* = 64–100 measurements/group). **f** Excessive mesangial expansion (white arrows) in WT-STZ mice at 24 weeks after diabetes onset. Scale bar, 0.5 µm. **g** Representative transmission electron micrographs of mitochondrial structure at 24 weeks after diabetes onset. Regular internal structure and elongated mitochondria (blue arrow), mitochondrial disrupted cristae (red arrow). **h** Quantitative analysis of mitochondrial area in each condition (*n* = 40 measurement/group). Scale bar, 0.5 µm. Results are expressed as fold over WT-Con. **i** Representative images show immunofluorescence staining to detect glomerular p57-positive podocytes (green) and nucleus (blue) at 24 weeks after diabetes onset. Scale bar, 50 µm. **j** Quantitative analysis of p57-positive podocytes at 24 weeks after diabetes onset (*n* = 30 glomeruli/group). Results are expressed as fold over WT-Con. **k** IHC staining for p57-positive podocytes (brown). Scale bar, 50 µm. One-way ANOVA with post hoc Tukey’s test for multiple comparisons. *P < 0.05, **P < 0.01, ***P < 0.001, ****P < 0.0001. All data are presented as mean ± SEM.

Glomerular lesions and early glomerular hypertrophy are among the most significant alterations in both early and late stages of DKD. As miR-379KO mice were protected from diabetes-induced albuminuria, to further define the relationship to renal histology, we prepared kidney cortical sections from STZ-injected and control-vehicle injected WT and miR-379KO mice. We used Periodic Acid–Schiff (PAS) staining to detect ECM accumulation and Masson’s Trichrome staining to assess kidney fibrosis. WT-STZ mice showed significant increase in mesangial matrix expansion compared to nondiabetic controls at 6 and 24 weeks of diabetes onset (Fig. 6a, b). Moderate glomerular tuft and tubulointerstitial fibrosis were observed in WT-STZ mice after 24 weeks of diabetes onset (Fig. 6c). Notably, all these increases related to DKD were ameliorated in miR-379KO-STZ mice (Fig. 6a–c). These results show that diabetic miR-379KO-STZ mice experience reduced severity in key features of early DKD, suggesting miR-379KO mice are protected from DKD.

### miR-379KO mice are protected from diabetes-associated GBM thickening and podocyte foot process effacement

We next used transmission electron microscopy (TEM) to examine glomerular structure in STZ- and control-treated WT and miR-379KO mice at 24 weeks after diabetes onset. Nondiabetic WT controls showed uniformly thin GBMs and normal structures of podocytes, and their foot processes; in contrast, WT-STZ mice exhibited thickening of the GBM and podocyte foot process effacement as expected (Fig. 6d). Nondiabetic miR-379KO mice also exhibited normal glomerular structures that were like WT control mice. However, the GBM thickening and podocyte foot process effacement observed in WT-STZ mice were abrogated in miR-379KO-STZ mice (Fig. 6d). Quantitative analysis confirmed that the GBM was significantly thinner in miR-379KO-STZ mice than in WT-STZ mice (Fig. 6e). Furthermore, WT-STZ mice developed excessive mesangial expansion with electron-dense deposits, which was attenuated in miR-379KO-STZ mice at 24 weeks after diabetes onset (Fig. 6f). Our TEM examination also showed the presence of abnormal mitochondrial structures and disrupted mitochondrial cristae in the glomeruli of WT-STZ mice, which were ameliorated in miR-379KO-STZ (Fig. 6g). Quantitative analysis also showed that the average mitochondrial area was smaller in WT-STZ mice compared to nondiabetic mice, but this reduction in area size was not observed in miR-379KO-STZ relative to corresponding nondiabetic mice (Fig. 6h).

Furthermore, a marker of podocytes (p57) was examined by immunofluorescence (IF) staining at 24 weeks after diabetes onset (Fig. 6i). The number of podocytes as detected by nuclear green fluorescent staining (p57) was lower in WT-STZ mice compared to WT Control, verifying podocyte loss in diabetic mice. Quantification of the IF data showed this podocyte loss was significantly attenuated in miR-379KO-STZ mice (Fig. 6j). IHC staining of p57 also showed decreased podocyte number in WT-STZ mice compared to WT-Con, which was reversed in the miR-379KO mice (Fig. 6k).

### Diabetes-induced increase in the expression of *Chop* and key cluster miRNAs are reduced in miR-379KO mice

**Fig. 7:**
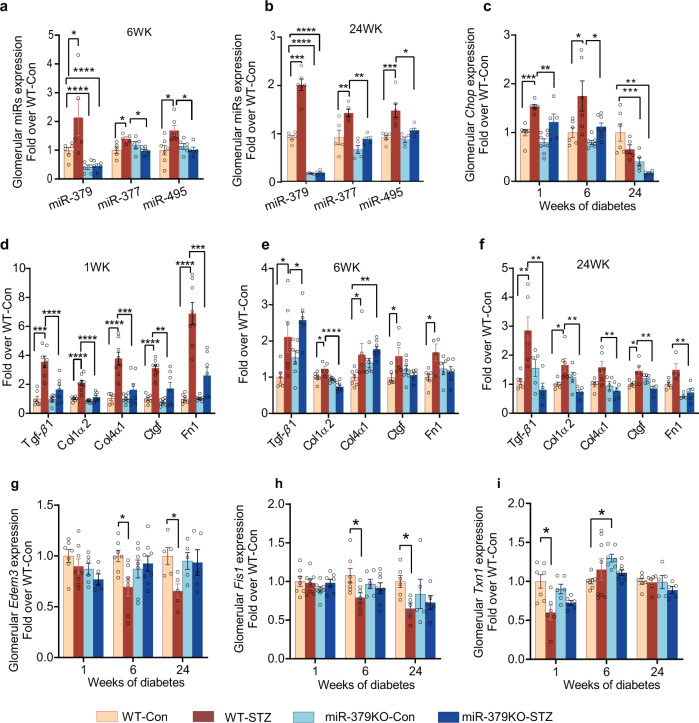
Glomerular expression of cluster miRNAs, profibrotic genes, and miR-379 target genes in WT and miR-379KO mice. **a**, **b** Expression of indicated miRNAs (miRs) in control and STZ-treated WT and miR-379KO mice at **a**, 6 and **b**, 24 weeks after diabetes onset (*n* = 4–6/group). **c** Expression of glomerular *Chop*, an ER stress-responsive transcription factor (*n* = 5–8/group, *n* = 6–8/group, and *n* = 5–6/group for 1, 6, and 24 weeks of diabetes, respectively). Results are expressed as fold over WT-Con, after normalization with internal control U6. **d**–**f** Glomerular expression of profibrotic genes, *Tgf-β1*, *Col1a2*, *Col4a1*, *Ctgf*, and *Fn1* at 1, 6, and 24 weeks (**d**–**f**) after diabetes onset (*n* = 5–8/group, *n* = 5–8/group, and *n* = 5–6/group for 1, 6, and 24 weeks of diabetes, respectively). **g**–**i** Glomerular expression of miR-379 target genes. **g** *Edem3*, **h** *Fis1*, and **i** *Txn1* were measured in WT and miR-379KO mice (*n* = 5–8/group, *n* = 6–8/group, and *n* = 5–6/group at 1, 6, and 24 weeks of diabetes, respectively). Results are expressed as fold over WT-Con, after normalization with internal control *Cypa*. Each dot indicates the value from each mouse. Statistical analyses were performed by one-way ANOVA with post hoc Tukey’s test for multiple comparisons. *P < 0.05, **P < 0.01, ***P < 0.001, ****P < 0.0001. All data are presented as mean ± SEM.

In our previous study, we found that miR-379 megacluster consists of about 40 other miRNAs besides miR-379, which is the first miRNA located at the 5′-end of the cluster^13^. Therefore, to determine whether loss of miR-379 affects some of the other downstream miRNAs in this cluster, we used real-time qPCR (RT-qPCR) to measure their expression in glomeruli isolated from STZ and control WT and miR-379KO mice. Compared to WT controls, in the glomeruli of WT-STZ mice, we found significant increases in the expression of candidate megacluster miRNAs, miR-379, miR-377, and miR-495. In contrast, these miRNAs were not increased in the glomeruli of diabetic miR-379KO-STZ mice at 6 (Fig. 7a) or 24 weeks (Fig. 7b), after the onset of diabetes.

We next examined the expression of *Chop*, which encodes the ER stress-responsive CHOP [CCAAT/enhancer-binding protein (C/EBP) homologous protein] that we previously identified to control the miR-379 megacluster and its host lncRNA^13^. We measured *Chop* gene expression in glomeruli isolated from STZ- and control-treated WT and miR-379KO mice. *Chop* expression was upregulated in the glomeruli at 1 and 6 weeks after diabetes onset but returned to normal levels at a later stage (24 weeks) (Fig. 7c). This diabetes-induced upregulation of *Chop* was however attenuated in miR-379KO-STZ compared to WT-STZ at 6 weeks and was even lower than normal at 24 weeks. This suggests that deletion of miR-379 may abrogate auto-upregulation of the miR-379 cluster in a unique fashion through the EDEM3–ER stress–CHOP axis^13^.

### Diabetes-induced expression of profibrotic genes is reduced in miR-379KO mice

To determine the role of profibrotic genes in the observed phenotype of miR-379KO-STZ mice showing protection from fibrosis and ECM accumulation, we next used RT-qPCR to measure the expression of *Tgf-β1*, collagens (*Col1α2* and *Col4α1*), connective tissue growth factor (*Ctgf*), and fibronectin (*Fn1*) in glomeruli isolated from STZ- and control-treated WT and miR-379KO mice. As expected, in WT-STZ mice, diabetes increased the expression of these profibrotic genes at 1, 6, and 24 weeks (Fig. 7d–f) after diabetes onset. This upregulation was significantly attenuated in miR-379KO-STZ mice at these time points (Fig. 7d–f). These results suggest that the genetic deletion of miR-379 attenuates glomerular fibrosis and ECM accumulation due to downregulation of key profibrotic genes in diabetic mice.

### Diabetes-induced reduction in the expression of miR-379 target genes is restored in miR-379KO mice

To determine putative connections between the key identified candidate miR-379 target genes and the phenotypes/gene expression seen in vivo in the diabetic miR-379KO-STZ mice, we used RT-qPCR to measure the expression of miR-379 target genes in glomeruli isolated from STZ- and control WT and miR-379KO mice. *Edem3* gene expression was significantly decreased in WT-STZ mice, but restored in miR-379KO-STZ mice, compared to their respective nondiabetic controls at 6 and 24 weeks after diabetes onset (Fig. 7g). These results are consistent with our previously published results, which showed decreased *Edem3* gene expression in STZ-induced and db/db diabetic mice^13^. In the current study, we identified *Fis1* and *Txn1* as new targets of miR-379. The expressions of both *Fis1* and *Txn1* were significantly decreased in WT-STZ mice, but not in miR-379KO-STZ mice, compared to their respective nondiabetic controls at 6 and 24 weeks after diabetes (for *Fis1*) and at 1 week post diabetes for *Txn1* (Fig. 7h, i). In general, the alterations in the levels of these targets were relatively mild and not statistically significant at some time points, likely because the glomeruli are heterogenous and include other cell types, such as podocytes and endothelial cells, besides mesangial cells.

**Fig. 8:**
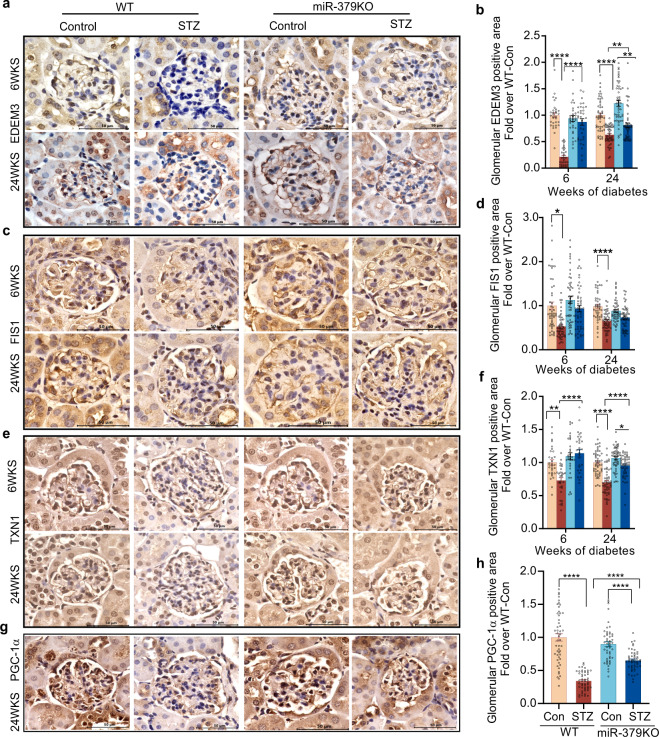
Immunohistochemical staining and quantitative analysis of miR-379 target proteins. **a**, **b** EDEM3 (*n* = 32 and 50 glomeruli/group for 6 and 24 weeks, respectively), **c**, **d** FIS1 (*n* = 50 glomeruli/group), **e**, **f** TXN1 (*n* = 30 and 50 glomeruli/group for 6 and 24 weeks, respectively), and **g**, **h** PGC-1a (*n* = 50 glomeruli/group) protein in kidney cortex sections from WT and miR-379KO mice at 6 and 24 weeks after diabetes onset. Scale bar, 50 µm. Bar graph results are expressed as fold over WT-Con. Statistical analyses were performed by one-way ANOVA with post hoc Tukey’s test for multiple comparisons. *P < 0.05, **P < 0.01, ****P < 0.0001. All data are presented as mean ± SEM.

To verify that the differential gene expression is accompanied by differential protein expression of these miR-379 targets in the glomerular compartments, we used IHC. We stained kidney cortex sections isolated from STZ- and control WT and miR-379KO mice with respective antibodies to detect EDEM3, FIS1, and TXN1 at 6 and 24 weeks after diabetes onset (Fig. 8). The average EDEM3-positive glomerular area was significantly smaller in WT-STZ mice compared to nondiabetic controls; this decrease was reversed in miR-379KO-STZ mice (Fig. 8a, b). FIS1-positive glomerular area also showed a significant decrease in WT-STZ mice, which was attenuated in miR-379KO-STZ mice (Fig. 8c, d). TXN1 staining was weaker in both the cytoplasm and nucleus in glomeruli of WT-STZ mice compared to WT nondiabetic mice, but significantly higher in miR-379KO-STZ than WT-STZ mice (Fig. 8e, f).

To further evaluate relations to mitochondrial function and biogenesis, we measured glomeruli peroxisomal proliferator-g coactivator-1a (PGC-1a) expression using IHC staining. PGC-1a is a major regulator or mitochondrial function and biogenesis. The results showed a significant decrease in glomerular PGC-1a levels in WT-STZ mice compared to nondiabetic controls. This reduction was significantly reversed in miR-379KO-STZ mice (Fig. 8g, h).

As we focused mainly on changes in glomeruli in vivo and in mesangial cells in vitro, to verify the above observed changes in these cells, we isolated MMC from the kidney glomeruli of normal mice and treated them with NG or HG as described in the “Methods” section. Then, cells were fixed and stained by IF with antibodies to EDEM3 and FIS1. Results from IF staining confirmed decreased expression of EDEM3 and FIS1 in the WT-MMC treated with HG vs. NG, but this decrease was clearly attenuated in miR-379KO-MMC. Notably, basal EDEM3 levels in miR-379KO MMC were significantly higher than WT MMC in NG conditions (Supplementary Fig. 7a–d).

To rule out the background and reveal nonspecific binding, normal kidney cortex sections were stained without primary antibodies for EDEM3, FIS1, TXN1, and PGC-1. No significant staining was detected without primary antibody (Supplementary Fig. 8).

Taken together, all these results indicate that deletion of miR-379 in mice plays a significant role in protecting renal glomerular cells (and possibly tubular cells) from mitochondrial dysfunction, ER and oxidative stress, and also in attenuating key features DKD progression, as well as weight loss in STZ-injected diabetic mice.

## Discussion

miR-379 is the most upstream miRNA in the miR-379 megacluster of miRNAs hosted by the lncRNA, lncMGC, which plays a role in early DKD^13^. In this study, we examined the in vivo role of miR-379 in the pathogenesis of DKD by studying the phenotype of miR-379KO mice, which we created using CRISPR-Cas9 editing. Although miR-379KO mice showed no apparent defects or detectable phenotypes under normal conditions, however, when rendered diabetic, they depicted protection from features of DKD, including albuminuria, glomerular hypertrophy, and fibrosis. It is interesting that the miR-379KO mice exhibited phenotypic variation only in response to a challenge (diabetes in this setting). This has also been seen in other single miRNA KO mice^31,32^, which may be because deletion of one miRNA does not critically affect embryonic or tissue development (due to compensation by other miRNAs/processes) but is however important for kidney disease progression likely due to functions of critical targets of the specific miRNA (miR-379).

**Fig. 9:**
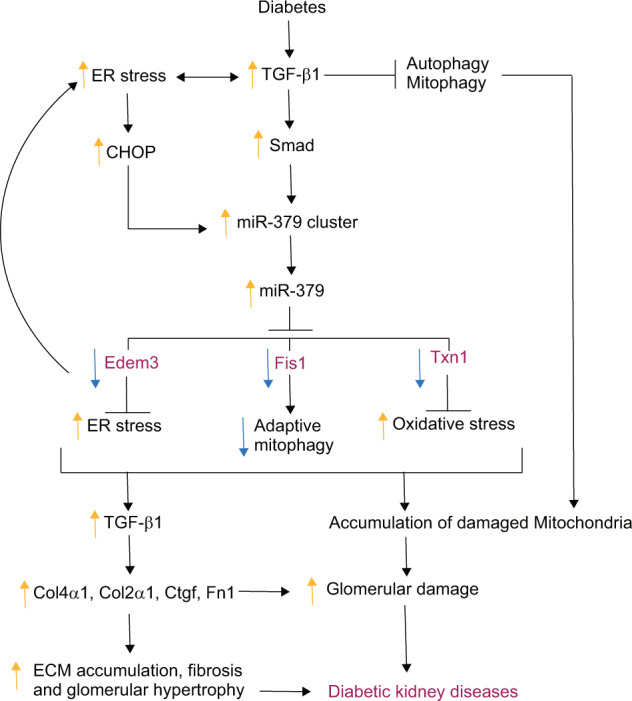
Proposed model illustrating the role of miR-379 in promoting early features of kidney disease in diabetic mice. Diabetic conditions induce expression of the miR-379 cluster via TGF-β signaling. This increase includes upregulation of miR-379 and subsequent downregulation of its targets (*Edem3*, *Fis1*, *Txn1*) related to ER stress, adaptive mitophagy, mitochondrial dysfunction, and oxidant stress, which are directly involved in the development of early features of DKD, such as ECM accumulation, glomerular hypertrophy, and fibrosis. Activated ER stress can also increase CHOP transcription factor and the miR-379 cluster miRNAs, creating a positive feedback. Genetic deletion of miR-379 can diminish these alterations induced by diabetes, interrupt the auto-feedback, and protect against DKD progression.

Using an AGO2-CLASH strategy in MMC from WT and miR-379KO mice, we identified several new targets of miR-379, while also confirming a previously identified target, *Edem3*^13^. We observed that the levels of key targets of miR-379 such as *Edem3*, *Fis1*, and *Txn1* were significantly reduced in WT diabetic mice compared to nondiabetic mice. However, their levels were restored in miR-379KO diabetic mice. EDEM3 is implicated in protection from ER stress^14^, FIS1 can promote adaptive mitophagy to preserve mitochondrial health^22^, whereas TXN1 is a known antioxidant^26,33^, which binds to and inactivates thioredoxin-interacting protein (TXNIP), a potent pro-oxidant. Thus, miR-379-mediated downregulation of these protective factors could be a mechanism by which miR-379KO mice are protected from DKD induced by ER stress, mitochondrial dysfunction, and oxidant stress (Fig. 9). These results are consistent with our previous report demonstrating that inhibition of lncMGC (host RNA of the miR-379 megacluster) by GapmeR antisense oligos reduced the expression of miR-379 and protected mice from early features of DKD through prevention of ER stress^13^. Our current study provides unequivocal evidence for the pathological role of endogenous miR-379. Importantly, we also demonstrate how miR-379 may mediate a pro-DKD phenotype by directly targeting genes such as *Fis1*, *Txn1* and *Edem3*.

We also mined the Nephroseq database (https://www.nephroseq.org) for the expression in human samples of miR-379 target genes identified from our CLASH profiling. Intriguingly we found that expressions of *EDEM3*, *FIS1*, and *TXN1*, as well as other candidate targets are significantly decreased in kidney samples from human subjects with diabetic and chronic kidney disease as well as other kidney diseases (Supplementary Figs. 9 and 10), suggesting that our results in diabetic mice are relevant to human chronic kidney disease.

Notably, our data suggests a protective role for FIS1 in DKD for the first time. FIS1 is involved in quality control of mitochondria via adaptive mitophagy pathways, in which FIS1 interacts with autophagy protein syntaxin17 to trigger mitophagy^25^. FIS1 displays different roles depending on which mitophagy pathway is involved^25^. In general, high levels of FIS1 triggers mitophagy^24^ and mutation of Fis1 disrupts mitophagy needed for elimination of damaged and dispensable mitochondria^23^ following cellular stress^21,34^. These results are consistent with our observations showing decreased expression of FIS1 in WT diabetic mice (and with HG) in parallel with impaired mitochondria quality. As autophagy is decreased in the diabetic kidneys^35–38^, mitochondrial dysfunction may be accelerated due to impaired clearance of damaged mitochondria as a result of reduced autophagy/mitophagy.

We also found that mitochondrial biogenesis, as assessed by TEM and expression of PGC-1α as surrogate marker, was impaired in the glomeruli of diabetic WT mice, but not in diabetic miR-379KO mice. PGC-1, a master transcriptional regulator of mitochondrial biogenesis and function, is decreased in diabetic kidneys^39^. Overexpression of PGC-1 can protect kidney fibrosis in chronic kidney disease^40^ and reduce oxidative stress in kidney cells in DKD^41,42^. We also observed that mitochondria-related genes were differentially enriched between WT and miR-379KO MMC. Furthermore, using Seahorse metabolic profiling, we observed a significant decrease in mitochondrial activity in WT MMC treated with HG, whereas miR-379KO MMC maintained higher mitochondrial activity even after HG treatment.

Mitochondrial quality and function are impaired in diabetic conditions, leading to increased generation of reactive oxygen species and oxidant stress associated with DKD^6,43,44^. TXN1 is a small multifunctional protein with oxidoreductase activity^26^. The antioxidant function of TXNIP is compromised by interaction with TXNIP and overexpression of TXN1 suppresses DKD in transgenic mice^45^. Our results showing lower expression of *Txn1* in the glomeruli of WT diabetic mice may be associated with increase in oxidant stress coupled with inflammation and upregulation of *Tgf-β1* in DKD, events that are reversed in miR-379KO mice. It is well-known that levels of TGF-β1, a master regulator of fibrosis, are increased in most renal cells in diabetes^1–5^. We also observed that increased expression of *Tgf-β1*, as well as other profibrotic genes such as *Col1αa2*, *Col4a1*, *Ctgf* and *Fn1* was attenuated in glomeruli from diabetic miR-379KO mice compared to diabetic WT mice. Diabetic conditions associated with increased *Tgf-β1* upregulate the expression of several miR-379 cluster miRNAs besides miR-379, as well as *Chop*, the gene encoding a well-known ER stress transcription factor^46^ and we found these factors are reduced in miR-379KO mice. These data suggest a feedback amplification loop, because our previous report showed CHOP upregulates the miR-379 cluster expression through XBP-1 splicing activation and reduction in ATF3 expression^3,13^. Of interest, we observed that, besides miR-379, other members of the miR-379 cluster showed lower expression in diabetic miR-379KO mice compared to WT mice. Genetic deletion of miR-379 may diminish such auto-upregulation of lncMGC, as well as the miR-379 cluster, thereby suppressing the expression of other members of the miR-379 cluster under diabetic conditions. miR-379KO mice are deficient in miR-379; therefore, they can be protected even in diabetic conditions, because miR-379 is not upregulated, and its targets (such as *Fis1*, *Edem3*, and *Txn1*) are not downregulated (or possibly even increased in the kidneys of miR-379KO mice).

Histological and TEM analyses showed that ECM accumulation, fibrosis, podocyte injury and loss, GBM thickness, and glomerular hypertrophy were all clearly reduced in diabetic miR-379KO compared to diabetic WT mice. Similar protection was previously observed in diabetic mice injected with a GapmeR targeting lncMGC^13^. Thus, our current study suggests that, in addition to ER stress regulated by the miR-379 target *Edem3*, mitochondrial dysfunction and oxidant stress regulated by the miR-379 targets *Fis1* and *Txn1* are also major players in DKD development (Fig. 9).

We also observed that WT diabetic mice depicted significant weight loss, which was not observed in miR-379KO. Body composition results suggest that recovery of lean mass in diabetic miR-379KO mice is the main reason for attenuated body weight loss, suggesting that mitochondrial dysfunction through miR-379-mediated decrease in Fis1 levels may be involved in muscle loss in WT diabetic mice but not in miR-379KO mice due, at least in part, to restoration of Fis1 levels. Future studies are however needed to systematically examine the role of miR-379 in skeletal muscle functions and parameters of atrophy in these mice.

As miR-379KO mice did not depict any overt defects or abnormal phenotypes under normal unchallenged conditions, inhibiting or reducing miR-379 is likely safe under normal healthy conditions. Under diabetic conditions, however, miR-379KO mice were protected from features of DKD. Thus, lowering miR-379 by targeting it directly, or its host lncMGC (with GapmeRs), could be an effective modality to slow down the progression of DKD.

## Methods

### Generation of miR-379KO mice

All animal studies were conducted according to protocols approved by the Institutional Animal Care and Use Committee at the Beckman Research Institute of City of Hope. Plasmids PX461 (enhanced GFP, EGFP) and PX462 (Puromycin)^15^ for gene editing with the CRISPR-Cas9 nickase strategy were obtained from Addgene. pSpCas9n(BB)-2A-GFP (PX461) were a gift from Feng Zhang, Broad Institute through Addgene (plasmid #48140; http://n2t.net/addgene:48140; RRID:Addgene;48140). pSpCas9n(BB)-2A-Puro (PX462) V2.0 was a gift from Dr. Feng Zhang (Addgene plasmid #62987; http://n2t.net/addgene:62987:62987; RRID:Addgene_62987). Double-stranded DNAs obtained by annealing of sense and antisense oligos (Supplementary Table 3) were cloned into BbsI-digested vectors^47^. Potential target sites (Supplementary Table 3) were designed using the online software CRISPRdirect^48^. For T7 in vitro transcription, we found that the pair of S2 and AS1 oligos (out of all pairs of S1, S2, or S3 and AS1 or AS2) worked best for in vitro screening (verified by deletion of miR-379 region by PCR, and reduction of miR-379 expression by RT-qPCR) using Transformed C3H Mouse Kidney-1 (TCMK1) cells,TCMK1 (ATCC, CCL-139). T7 priming site-tagged PCR fragments were amplified from the plasmids using primers T7S2 5′-TTAATACGACTCACTATAGGGATGGTAGACTATGGAACGT-3′, T7AS1 5′-TAATACGACTCACTATAGGGTTAGTGGACCATGTTACAT-3′, and guide RNA-R 5′-AAAGCACCGACTCGGTGCC-3′.

Amplified PCR fragments were used as templates for in vitro transcription using a T7 in vitro transcription kit (MEGAscript TM T7 Transcription Kit, Ambion). In vitro transcribed RNAs were purified using MEGAclear™ Transcription Clean-Up Kit (Ambion). Guide RNAs and Cas9 nickase RNA (TriLink BioTechnologies) were injected into fertilized eggs from female C57BL/6J mice to produce mutant mice. A mixture of S1 sgRNAs, AS2 sgRNAs (50 ng/μl total) and 50 ng/μl Cas9 nickase mRNA was microinjected into the cytoplasm of fertilized C57BL/6J 1-cell embryos using standard methods and these embryos were implanted surgically into pseudo-pregnant recipient female mice to produce mutant mice.

Mutant candidate mice were screened for the anticipated 36 bp deletion by performing PCR with DNA extracted from the tails of surviving mice. Several founders had shorter fragments relative to WT by fragment analysis^49^ and the deletion was confirmed by sequencing (Fig. 1b). Founders confirmed to have the anticipated 36-bp deletion were crossed with WT C57BL/6 mice and subsequent litters tested for germline transmission of the miR-379 deletion. Heterozygotes were crossed with each other to obtain homozygotes. Significant decrease of miR-379 expression was confirmed using qPCR in miR-379KO mice.

### Mouse models of diabetes

Diabetes was induced by injecting 50 mg/kg STZ intraperitoneally daily for 5 consecutive days in 10-week-old male WT (WT-STZ) and miR-379KO (miR-379KO-STZ) C57BL/6 mice in accordance with the Diabetes Complications Consortium (https://www.diacomp.org/). Male mice injected with diluent (citrate buffer pH 4.0) served as controls (WT-Con and miR-379KO-Con). Diabetes was confirmed by measuring tail vein blood glucose levels (fasting glucose > 300 mg/dl). As, unlike male mice, STZ-injected female mice of the same age did not develop high blood glucose levels, female mice were not evaluated in this study. Each experimental group was composed of five to eight mice. Mice were killed at 1, 6, and 24 weeks post-induction of diabetes. After killing, blood was taken from the heart for serum collection, and kidneys were harvested for isolation of glomeruli, preparation of MMC, and histopathological examination (Supplementary Fig. 5).

### Isolation of mouse glomeruli and preparation of primary MMCs

Glomeruli were isolated from freshly harvested mouse kidneys using an established sieving technique^13^. Briefly, renal capsules were removed, and the cortical tissue of each kidney was separated by dissection. The cortical tissue was then carefully strained through a stainless sieve with a pore size of 200 μm by applying gentle pressure. Enriched glomerular tissue below the sieve was collected and transferred to another sieve with a pore size of 150 μm and then 75 μm. After several washes with cold PBS, the glomeruli remaining on top of the sieve were collected. Pooled glomeruli were centrifuged and the pellet was collected for RNA, protein extraction, or for preparing MMC according to our reported methods^13^. MMC were maintained in Roswell Park Memorial Institute medium 1640 medium supplemented with 10% fetal bovine serum. Passages 5–7 were used for experiments^13^.

### RNA sequencing

Total RNA was subjected to library preparation using the KAPA Stranded mRNA-Seq Kit (KK8421; Kapa Biosystems) followed by cluster generation and sequencing performed on HiSeq 2500 platform with 51 bp single-end reads or 101 bp paired-end reads at City of Hope’s Integrative Genomics Core.

### RNA-seq data analysis

Raw sequences were aligned to the mouse reference genome mm10 using TopHat v2.0.14^50^ with default parameters, and gene-level expression levels of all the mouse RefSeq genes (downloaded from USCS genome Browser, https://genome.ucsc.edu) were counted using htseq-count^51^ v 0.6.0 with settings -s reverse (strand-specific) and -a 10 (filtrating poor alignment reads). The counts were normalized by trimmed mean of M value (TMM) method^52^ and differentially expressed genes between groups were identified using Bioconductor package edgeR^53^ (fold change > 1.5 and *P* < 0.05). IPA was applied to differentially expressed genes for gene ontology and pathway analyses.

### AGO2-CLASH technique

CLASH procedures were performed according to published methods^19,20^. Briefly, MMC from WT and miR-379KO mice were plated in four 10 cm petri dishes (70% confluence), washed with PBS, and cross-linked by exposure to 50 J/m^2^ UV using a UV Stratalinker (Stratagene). RNA was isolated using a miRNA Isolation kit (mouse AGO2) obtained from WAKO, Japan (Catalog number 292-67301). Briefly, cross-linked cells were collected using 200 μl lysis buffer (provided in the miRNA Isolation kit, WAKO) per dish, gently mixed, incubated on ice for 10 min, sonicated (30 s × 5 with 30 s intervals) using a BIORUPTOR (Diagenode), and stored at −80 °C. AGO2 RISC complex was immunoprecipitated with anti-mouse AGO2 beads (WAKO) by gently rocking at 4 °C and washed with lysis buffer three times. The 5′-ends of RNAs in the immunoprecipitates were phosphorylated using polynucleotide kinase PNK (New England BioLabs (NEB), Ipswich, MA) (50 units in 20 μl) at 20 °C for 2 h, washed with lysis buffer, ligated using T4 RNA ligase (NEB) (50 units in 20 μl) at 16 °C for 16–18 h (overnight), eluted, extracted using phenol-isoamyl alcohol and chloroform, precipitated with ethanol, washed with 70% ethanol, air dried, and dissolved in 10 μl nuclease-free water. Extracted RNAs were subjected to next-generation RNA-seq or conventional qPCR. To detect enrichment of potential target RNAs in AGO2 RISC, RNAs extracted without ligation reaction from WT and miR-379-KO MMC were also sequenced (small RNA-seq) and compared to the enrichment of RNAs between WT and miR-379-KO MMC.

#### CLASH sequencing

To detect miRNA–mRNA hybrid reads, libraries were constructed based on Illumina’s small RNA-seq protocol as described previously^13^, but without size selection. Specifically, 5 μl of crossed-linked RNA was directly ligated to 3′-adapter (5′-UCUGGAAUUCUCGGGUGCCAAGGAACUCC-3′) followed by 5′-adapter (5′-GUUCAGAGUUCUACAGUCCGACGAUC-3′), reversed-transcribed to single-stranded cDNA, and amplified by PCR. The constructed library was then undergone purification, cluster generation and 101 bp paired-end sequencing on HiSeq 2500 platform by the same core.

#### CLASH-seq data analysis

Paired-end raw sequenced reads were merged using pear v0.9.5 with parameters settings -n 30 -p 1.0. Any merged reads aligned to either mouse whole genome (GRCm38.p5) or RNA transcripts (GENCODE vM13) using Bowtie2 v2.1.0 with settings -very-sensitive were subsequently removed. The remaining merged reads were then searched to identify the ones partially aligned to mature miRNA (miRbase release 21) and/or mRNA sequences (GENCODE vM13) using blastn program in blastall v2.2.26 with parameters (-e 10 -S 3 -W 7 -m 8 for miRNA and -e 0.1 -S 3 -W 7 -m 8 for mRNA). Finally, chimeric reads were selected as the merged reads if (1) they are partially aligned to any mature miRNA and any mRNA and (2) the aligned regions are not overlapped. The chimeric reads containing partial mature miR-379 sequences were further identified.

#### AGO2-IP RNA-seq data analysis

For each sample, paired-end 101 bp raw data were processed using the same approach as done on single-end samples (aligned by TopHat, counted by HTseq-count, TMM-normalized). Difference on expression between KO vs. WT samples were performed using simple model in edgeR. To identify miR-379 targets in mouse, 94 potential targets (genomic locations) on mm10 predicted by miRanda/mirSVR were first obtained from starBase v2.0^54^ (http://starbase.sysu.edu.cn/starbase2/targetSite.php). The 5′-end of each target was then extended to 100 bp in width. For each target, read counts located in both the extended target region (TAR) and non-target 3′-UTR region (NT3UTR) were summarized using R packages “rtracklayer” and “GenomicRanges”. The local enrichment of each TAR relative to its own NT3UTR region was calculated using log2 (TAR/NT3UTR) in each sample. Enrichment level between IPKO (Immuno Precipitation Knock-Out) and IPWT (Immuno Precipitation Wild-Type) samples at each target with local enrichment level < 1 was compared and targets were ranked based on the fold change between KO and WT samples.

### miRNA mimics and siRNAs

Oligonucleotide mimics of miRNAs, siRNAs, and corresponding control oligos were obtained from Integrated DNA Technologies or Thermo Fisher Scientific, Inc. (Waltham, MA), as described^55,56^. Briefly, MMC (~10^6^/transfection) were transfected with siRNA or miRNA oligos using an Amaxa Nucleofector (Lonza, Basel, Switzerland) according to the manufacturer’s protocols as described previously^55,56^. Mouse Fis1 siRNAs (double-stranded oligos were obtained from Thermo Fisher Scientific, Inc. Non-targeting siRNA controls were obtained from Thermo Fisher Scientific, Inc. MMC were trypsinized and resuspended in Basic Nucleofection Solution at 1 × 10^7^/ml. Subsequently, 100 μl of cell suspension (1 × 10^6^ cells) was mixed with miRNA mimic, inhibitor oligonucleotides, or ON-TARGET plus siRNA or NCs (Thermo Fischer Scientific, Inc., Waltham, MA). Transfected cells were collected for RNA extraction or fixed for IHC staining. RNA was extracted from the cells and the expression of miRNAs within the cluster was examined using primers designed for each of the mature miRNAs (miRBase).

### Luciferase reporter assays

Mouse *Fis1* 3′-UTR and *Txn1* 3′-UTR regions were amplified by PCR using the primers shown below (F and R refer to forward and reverse primers, respectively): mFis1 3UTR F-XhoI 5′-gagctcgAGGCAGCCTCACCTGCTCTC-3′, mFis1 3UTR R-NotI 5′-cctgcggccgcACACTCATTGCAAAGCATGACAGG-3′; m*Txn1*-3UTR F-XhoI 5′-TGctcgagTCATGCTCTGAAAAGTGTAACCA-3′, m*Txn1*-3UTR R-NotI 5′-CTgcggccgcTCATTTATATTAAAACACATCAGGCAG-3′.

Amplified *Fis1* 3′-UTR and *Txn1* 3′-UTR were digested with XhoI and NotI, and then cloned into the *Not*I-*Xho*I site of the psiCheck2 vector (Promega). For mutagenesis of the miR-379-binding site in *Fis1* 3′-UTR (designated MT for mutant), the primers listed below were used.

Mutagenesis primers: m*Fis1* 3UTR MT-F 5′-CACCCCTGTAGgagctCTCTACAGTCT-3′ and m*Fis1* 3UTR MT-R, 5′-AGACTGTAGAGAGCTCCTACAGGGGTG-3′, where F and R refer to forward and reverse primers, respectively. Lower cases are mutated bases.

For mutagenesis of the miR-379-binding site in the *Txn* 3′-UTR (designated MT), PCR fragment including deletion of miR-379-binding site was amplified using primers below and cloned into psiCheck2. m*Txn1*-3UTR-F-XhoI 5′-TGctcgagTCATGCTCTGAAAAGTGTAACCA-3′ and m*Txn1*-3UTR-379del-R-NotI 5′-CAgcggccgcTTAATATTTTATTGTCATTTATAATCAGATG-3′. Lower cases are restriction enzyme sites for cloning into plasmid vectors.

MMC were transfected with these reporter plasmids along with miR-379 mimic or NC oligos (Thermo Fischer Scientific, Inc., Waltham, MA) using an Amaxa Nucleofector. Luciferase activity was measured and analyzed according to manufacturer’s protocol, as described previously^56^.

### Real-time qPCR

RT-qPCR analysis was performed as previously described^13^. Briefly, RNA was extracted using an RNEASY Mini kit (Qiagen, Valencia, CA). miRNA quantification was performed using the qScript miRNA cDNA synthesis kit (Quanta Biosciences, Gaithersburg, MD) and amplified using PerfeCTa SYBR Green Supermix (Quanta Biosciences). For miRNAs, specific mature miRNA sequences were used as forward primers and the universal primer provided in the kit as the reverse primer. U6 was used as an internal control. A GeneAmp RNA PCR kit (Applied Biosystems, Carlsbad, CA) and POWER SYBR Green mix (Applied Biosystems) were used for mRNA quantification. mRNA expression normalized to Cypa as an internal control. PCR primer sequences are shown in Supplementary Table 4.

### Measurement of body composition and metabolic parameters

Whole body composition (fat and lean tissues) of mice was determined using quantitative magnetic resonance imaging technology (EchoMRI™ 3-in-1; Echo Medical Systems, Houston, TX). Automatic tuning and calibration of the instrument parameters using canola oil maintained at room temperature (22 °C) were performed daily. One mouse was placed in the analytical chamber at a time. Parameters measured by the EchoMRI™ included lean mass (g), fat mass (g), free water (g), and total water (g). Alterations to body composition metabolic parameters such as VO2, VCO2, RER, food intake, water consumption, and locomotor activity were also measured using an indirect calorimetry cage system (PhenoMaster, TSE Systems, Bad Homburg, Germany) located in the City of Hope Comprehensive Metabolic Phenotyping core.

Mice were weighed and then individually housed in cages for 3 days. Animals were acclimated to the cages for 1 day and experimental data were collected for 48 h following the acclimation day. Animals were given ad libitum access to food and water and housed in a temperature-controlled environment with a 12 h light/dark cycle for the duration of the experiment. The PhenoMaster is an indirect calorimetry system with gas-sensing units to measure VO_2_ and VCO_2_. A multi-dimensional infrared beam system allows the measurement of locomotor activity, which was defined as the total number of infrared beam breaks in the *X* and *Y* axis. The software was set to divide the experimental period into 30 min segments.

### Metabolic flux assays and mitochondrial respiration assays

OCR and extracellular acidification rate were measured using Agilent Seahorse XF Cell Mito Stress Test Kit on an XF96 Extracellular Flux Analyzer (Agilent, Santa Clara, California, USA). MMC were seeded at a density of 6000–8000 cells per well in 96-well plates. Cells were treated with NG (5.5 mM) and HG (25 mM) for 72 h before switching to Dulbecco’s modified Eagle’s medium base medium pH 7.4 (supplemented with 10 mM glucose, 1 mM sodium pyruvate, and 2 mM glutamine) on the day of the assay and incubated at 37 °C for 1 h in a non-CO_2_ incubator. Mitochondrial function parameters were determined following serial addition of three mitochondrial inhibitors, ATP synthase inhibitor oligomycin (2 mM final concentration), proton carbonyl cyanide *p*-trifluoromethoxyphenylhydrazone (1.5 mM final concentration), and a mixture of complex I and III inhibitors, rotenone, and antimycin A, respectively (0.5 mM each final concentration). Mitochondrial function was assessed by determining the basal respiration, ATP turnover rate, proton leak, and maximal and spare respiratory capacity using the mitochondrial inhibitors described above. OCR was calculated using Seahorse XF96 analyzer software.

### Method to assess mitochondrial quality and adaptive mitophagy

For detection of mitochondria by fluorescence microscopy, WT and miR-379KO MMCs were transfected with the DsRed2-Mito-7 plasmid (Addgene plasmid# 55838), which fluorescently labels mitochondria with red emission spectra. For quantification analysis, all images were captured at ×60 magnification using a Keyence microscope (KEYENCE-BZ-X800 Series, Osaka, Japan) and quantitatively analyzed using ImageJ software (ImageJ. win32). To measure the mean fluorescence intensity of labeled mitochondria in each cell, we used a previously published method^57,58^, based on integrated density, called “Corrected Total Cell Fluorescence (CTCF).” This method normalizes the mean intensity of each cell area to the background using the following formula: CTCF = Integrated Density − (Area of selected cell × Mean fluorescence of background readings).

To monitor adaptive mitophagy, MMCs were transfected with pCLBW-cox8-EGFP-mCherry (also known as addgene #78520)^23^. WT or miR-379KO MMC were then incubated in HG (25 mM) or NG conditions at 37 °C and 5% CO_2_ for 5 days, and the experiments were repeated three times. For live-cell imaging, the live cells were placed in the environmental chamber of Keyence microscope (KEYENCE.BZ-X800E), which is maintained at 37 °C and 5% CO_2_. The 488 nm and 561 nm were used to excite cox8-EGFP-mCherry. In these experiments, as described in the “Results,” normal cells with healthy mitochondria expressed yellow signals (i.e., green plus red fluorescence), whereas mitochondria depicting adaptive mitophagy showed predominantly red fluorescence, due to the selective sensitivity of EGFP fluorescence to low pH. All images were captured at ×60 magnification. The number of mitochondria within each cell was counted using ImageJ software (ImageJ. win32) and then the percentage of red-only puncta in cells was calculated.

### Measurement of urine albumin and creatinine concentration in mice

Albumin was measured as a marker for kidney damage and dysfunction in 24-h collected urine samples using an ELISA kit (Crystal Chem#80630). Urine creatinine was measured using a high-quality enzymatic assay for mouse creatinine (Crystal Chem#80350). Urine albumin excretion was then normalized to urine creatinine to obtain ACRs to correct for variations in urine flow rate.

### Histology and IHC

Kidney cortex samples were fixed in 10% formalin and prepared for light microscopic examination according to routine histological techniques (Pathology: Solid Tumor Core, City of Hope). The sections were stained with PAS to identify ECM accumulation and with Masson’s Trichrome for detection of fibrosis in the glomerular mesangial area. Under light microscopy examination, glomerular size, mesangial PAS-positive areas, and cortex fibrotic areas were measured in each group using Image-Pro Premier 9.2 software.

The cellular localization of EDEM3 (1:100, NBP1-88342, Novus Biologicals), FIS1 (1:250, ab71498, Abcam), TXN1 (1:400, ab86255, Abcam), p57 (1:500, ab75974, Abcam), PGC-1α (1:300, ab54481, Abcam), ATG5 (1:150, ab108327, Abcam), and p62 (1:100, ab155686, Abcam) was examined in glomerular sections using IHC. Biotinylated goat anti-rabbit (1:400, BA-1000, Invitrogen) was used as secondary antibody. To rule out the background by the secondary antibody, we performed control staining without primary antibody using normal mouse kidney sections followed with incubation with Avidin : Biotinylated enzyme Complex (ABC) system (VECTASTAIN ABC Kits-Vector) and then DAB (Supplementary Fig. 8). IHC images were taken at ×20 magnification and immune-positive reaction areas were quantified in the glomeruli in each group using ImageJ software.

IF staining was performed to show the cellular localization of p57^59,60^, podocyte marker (1:250, ab75974, Abcam), EDEM3 (1:100, NBP1-88342, Novus Biologicals), and FIS1 (1:250, ab71498, Abcam), and with the following Alexa Fluor 488, goat anti-rabbit (1:500, A11034, Invitrogen) secondary antibody. IF images were taken using original magnification of ×20. p57-positive cells were counted in 30 glomeruli from each group using BZ-X800 image analyzer (KEYENCE-BZ-X800 Series, Osaka, Japan). EDEM3- and FIS1-specific fluorescence (integrated density) were measured (*n* = 15–20 cell/group) using ImageJ software (ImageJ-Win32) and then CTCF was calculated as described above.

### TEM examination

The cortices of the mouse kidney were fixed in 2.5% v/v glutaraldehyde solution for 24 h, then processed for TEM examination according to routine TEM processing and staining protocols for tissues in the City of Hope Electron Microscopy and Atomic Force Microscopy Core^13^. Ultra-thin sections were cut using a Leica Ultra-cut UCT ultramicrotome. TEM was performed on FEI Tecnai 12 TEM equipped with a Gatan Ultrascan 2 K CCD camera. Average GBM thickness was quantified in ~100 measurements and area of 40 mitochondrion was measured in each group using Image-Pro Premier 9.2 software.

### Statistics and reproducibility

Statistical data analyses were performed using GraphPad Prism software (8.2.1). Normal distribution of each sample group was confirmed using *χ*^2^-test or Shapiro–Wilk test before comparison between groups. All data were expressed as mean ± SEM and statistical analyses were performed using Student’s *t*-tests (two-sided) to compare two groups or analysis of variance followed by post hoc Tukey’s test, to compare multiple groups. For all experiments, the number of replicates is shown in the figure legends. For experiments with mice, the number included is also noted in the figure legends. A minimum sample size of five mice in each group was used based on both our previous experience and power analyses. We have at least 80% power to detect an effect size of two between WT and KO groups, or diabetic and nondiabetic groups at a confidence level *p* < 0.05 using two-sided *t*-tests. Asterisks indicate significant difference (*p < 0.05, **p < 0.01, ***p < 0.001, and ****p < 0.0001).

## Supplementary information

Supplementary Information

Description of Additional Supplementary Files

Supplementary Data 1

Supplementary Data 2

## Data Availability

All sequencing datasets generated in this study have been deposited into GEO with GEO #s GSE142596, GSE142597, and GSE142598. The source data underlying the graphs in figures are provided in Supplementary Data 1 and 2. Full gel is shown in Supplementary Information. All relevant data are available from the authors upon request.
