# Publisher Correction: miR-379 deletion ameliorates features of diabetic kidney disease by enhancing adaptive mitophagy via FIS1

**DOI:** 10.1038/s42003-021-01691-4

**Published:** 2021-02-04

**Authors:** Mitsuo Kato, Maryam Abdollahi, Ragadeepthi Tunduguru, Walter Tsark, Zhuo Chen, Xiwei Wu, Jinhui Wang, Zhen Bouman Chen, Feng-Mao Lin, Linda Lanting, Mei Wang, Janice Huss, Patrick T. Fueger, David Chan, Rama Natarajan

**Affiliations:** 1 Department of Diabetes Complications and Metabolism, Diabetes and Metabolism Research Institute, Beckman Research Institute of City of Hope, 1500 East Duarte Road, Duarte, CA 91010 USA; 2 Transgenic Mouse Facility, Center for Comparative Medicine, Beckman Research Institute of City of Hope, 1500 East Duarte Road, Duarte, CA 91010 USA; 3 Integrative Genomics Core, Beckman Research Institute of City of Hope, 1500 East Duarte Road, Duarte, CA 91010 USA; 4 Irell and Manella Graduate School of Biological Sciences, Beckman Research Institute of City of Hope, 1500 East Duarte Road, Duarte, CA 91010 USA; 5 Department of Cellular and Molecular Endocrinology, Beckman Research Institute of City of Hope, 1500 East Duarte Road, Duarte, CA 91010 USA; 6 Comprehensive Metabolic Phenotyping Core, Beckman Research Institute of City of Hope, 1500 East Duarte Road, Duarte, CA 91010 USA; 7 Division of Biology and Biological Engineering, Caltech, 1200 East California Boulevard, Pasadena, CA 91125 USA

**Keywords:** End-stage renal disease, Genetic engineering

Correction to: *Communications Biology* 10.1038/s42003-020-01516-w, published online 4 January 2021.

In the original version of the published Article, the addresses for affiliations 1–5 were incorrect. The correct seven affiliations are listed below, along with the authors who belong to each affiliation:

Affiliations

1. Department of Diabetes Complications and Metabolism, Diabetes and Metabolism Research Institute, Beckman Research Institute of City of Hope, 1500 East Duarte Road, Duarte, CA 91010, USA

Mitsuo Kato, Maryam Abdollahi, Ragadeepthi Tunduguru, Zhuo Chen, Zhen Bouman Chen, Feng-Mao Lin, Linda Lanting, Mei Wang and Rama Natarajan

2. Transgenic Mouse Facility, Center for Comparative Medicine, Beckman Research Institute of City of Hope, 1500 East Duarte Road, Duarte, CA 91010, USA

Walter Tsark

3. Integrative Genomics Core, Beckman Research Institute of City of Hope, 1500 East Duarte Road, Duarte, CA 91010, USA

Xiwei Wu and Jinhui Wang

4. Irell and Manella Graduate School of Biological Sciences, Beckman Research Institute of City of Hope, 1500 East Duarte Road, Duarte, CA 91010, USA

Zhen Bouman Chen and Rama Natarajan

5. Department of Cellular and Molecular Endocrinology, Beckman Research Institute of City of Hope, 1500 East Duarte Road, Duarte, CA 91010, USA

Janice Huss and Patrick T. Fueger

6. Comprehensive Metabolic Phenotyping Core, Beckman Research Institute of City of Hope, 1500 East Duarte Road, Duarte, CA 91010, USA

Patrick T. Fueger

7. Division of Biology and Biological Engineering, Caltech, 1200 East California Boulevard, Pasadena, CA 91125, USA

David Chan

