# Supplementary material for: miR-379 deletion ameliorates features of diabetic kidney disease by enhancing adaptive mitophagy via FIS1
Source: Commun Biol. 2021 Jan 4;4:30. doi: 10.1038/s42003-020-01516-w (PMC7782535; doi:10.1038/s42003-020-01516-w)
Supplement: Supplementary file 1 — Supplementary Information [file 42003_2020_1516_MOESM1_ESM.pdf]

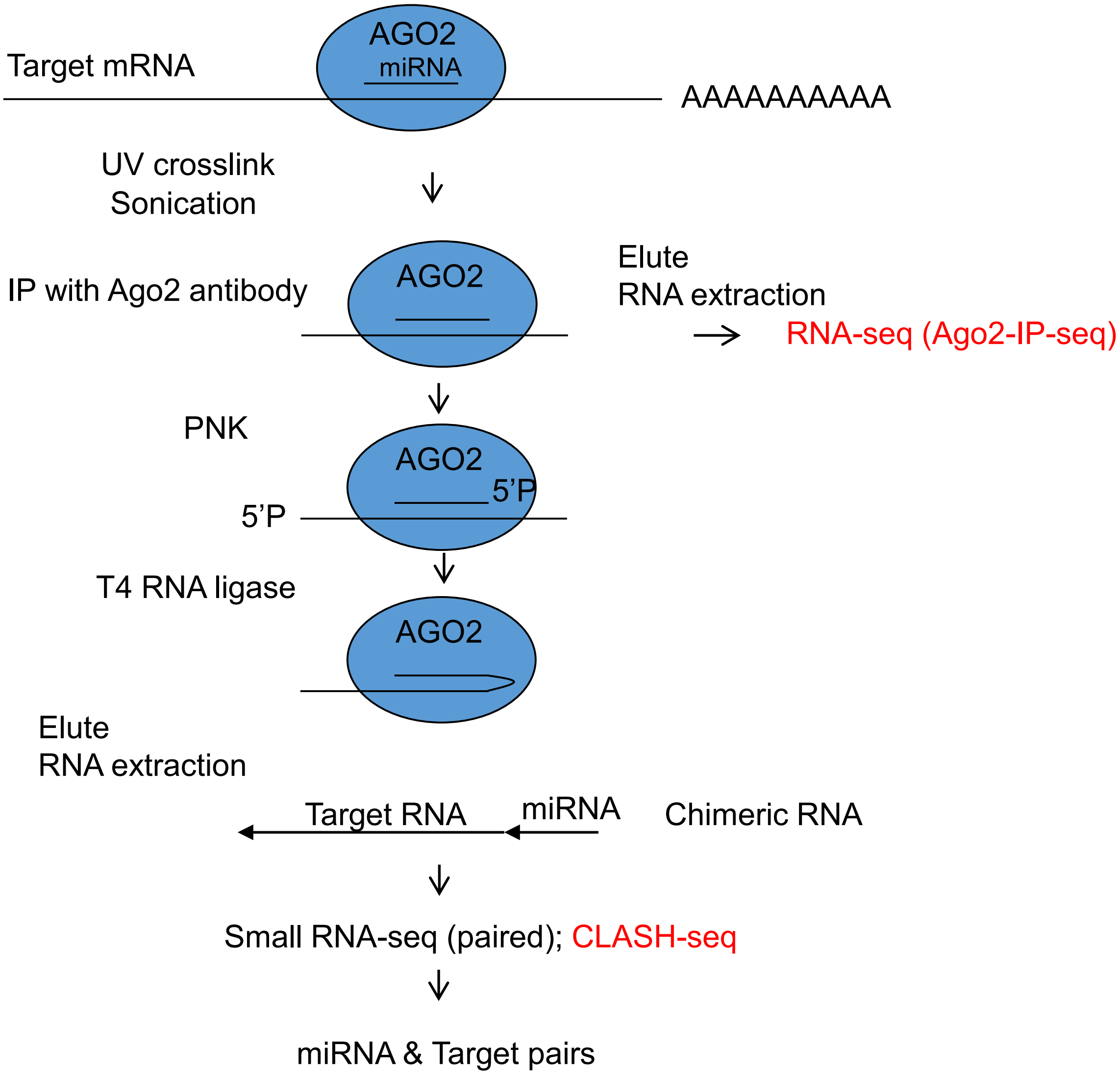

Supplementary Fig. 1 | Identification of miR-379 targets by AGO2-CLASH method. **a,** Schematic flow through of CLASH procedure. MMC from WT and miR-379KO mice were plated in four 10-cm petri dishes (70% confluence), washed with PBS, and cross-linked by exposure to 50J/m2 UV. Cross-linked cells were harvested and sonicated. AGO2 RISC complex was immunoprecipitated with anti-mouse AGO2 beads and washed with lysis buffer 3 times. The 5' ends of RNAs in precipitates were phosphorylated using polynucleotide kinase (PNK), washed with lysis buffer, ligated using T4 RNA ligase, eluted, extracted using phenol-isoamylalcohol and chloroform, precipitated with ethanol, washed with 70% ethanol, air dried, and dissolved in 10 μl nuclease-free water. Extracted RNAs were subjected to next-generation RNA-seq or conventional qPCR. To detect enrichment of potential target RNAs in AGO2 RISC, RNAs extracted without ligation reaction were also sequenced and compared to RNA-seq results with ligation.

.

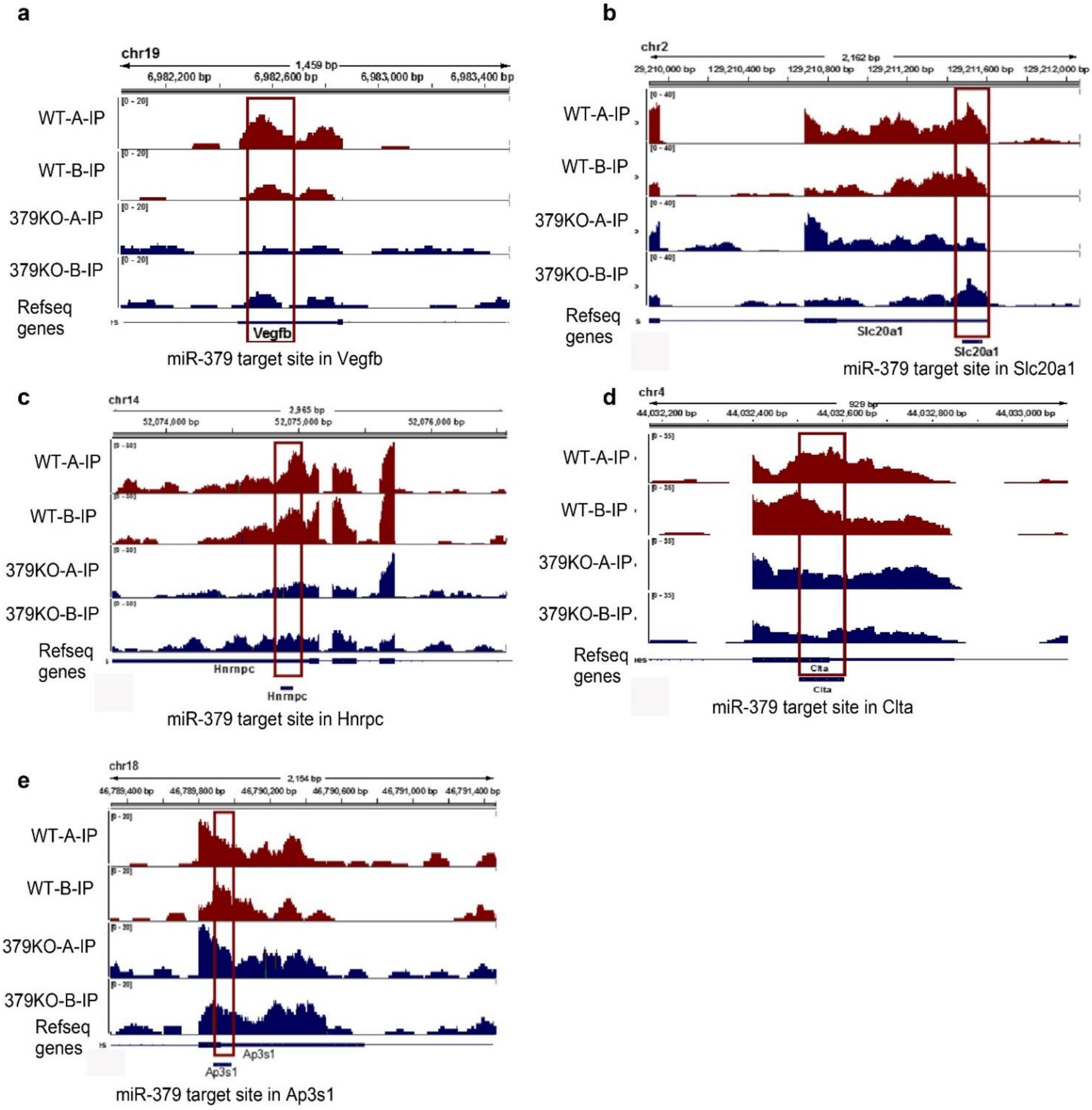

Supplementary Fig. 2. | To detect enrichment of potential target RNAs in AGO2 RISC, RNAs extracted without ligation reaction were also sequenced and compared to RNA sequencing results with ligation. Enrichment of RNA reads at miR-379 target site of **a**, *Vegfb* **b**, *Slc20a1* **c**, *Hnrnpc* **d**, *Clta* and **e**, *Ap3s1* 3'UTR in WT MMC, but significant reduction in miR-379KO MMC. Two independent samples (A and B) from WT MMC (WT-A-IP and WT-B-IP) and miR-379KO MMC (379KO-A-IP and 379 KO-B-IP) were examined

a

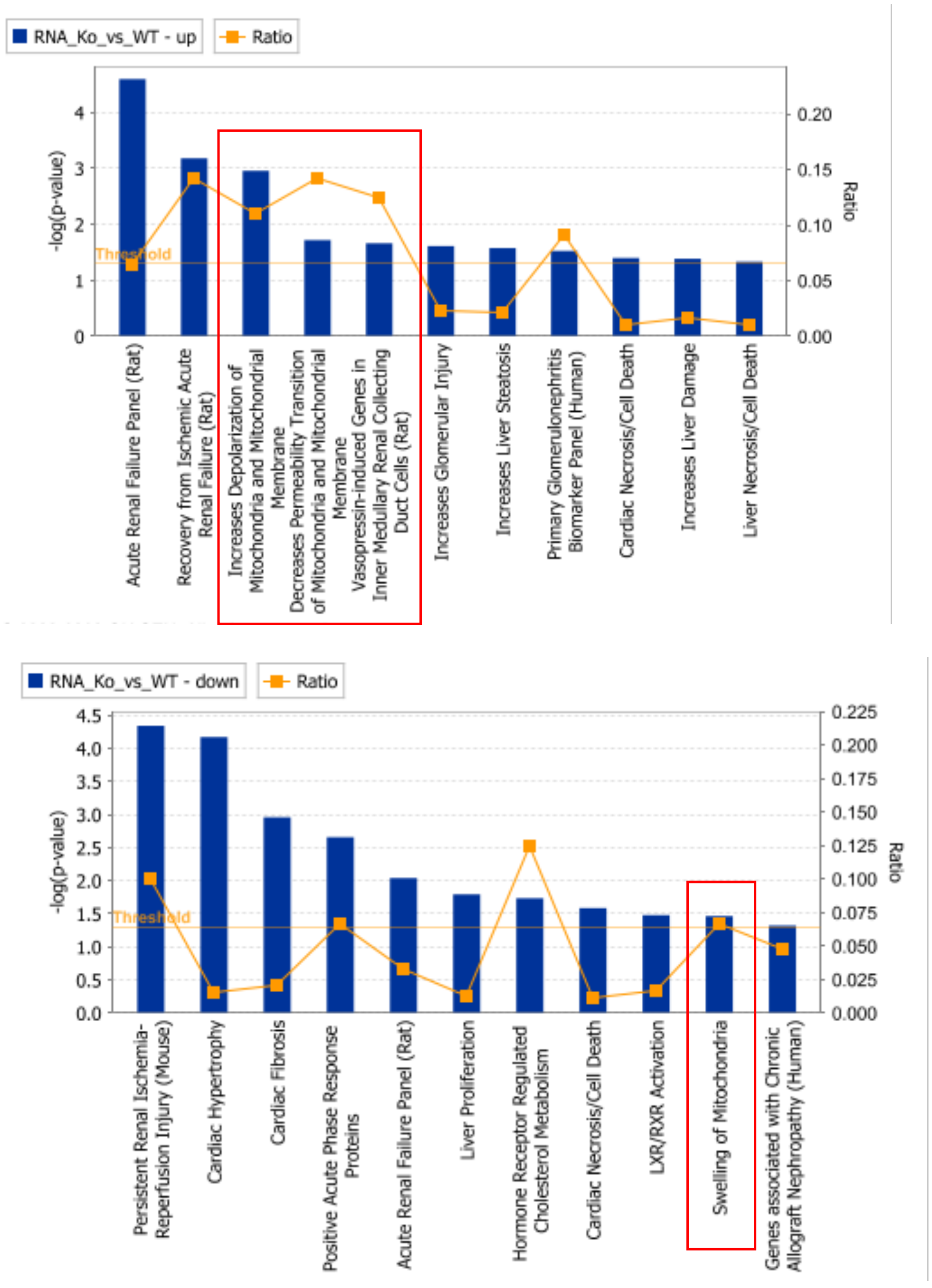

b

Supplementary Fig. 3 | Pathway analysis of differentially expressed genes in miR-KO MMC versus WT MMC using RNA-seq data. **a**, Upregulated pathways in miR-379KO MMC compared to WT MMC. **b**, Downregulated pathways in miR-379KO MMC compared to WT MMC. Both suggest the involvement of pathways related to mitochondrial activity.

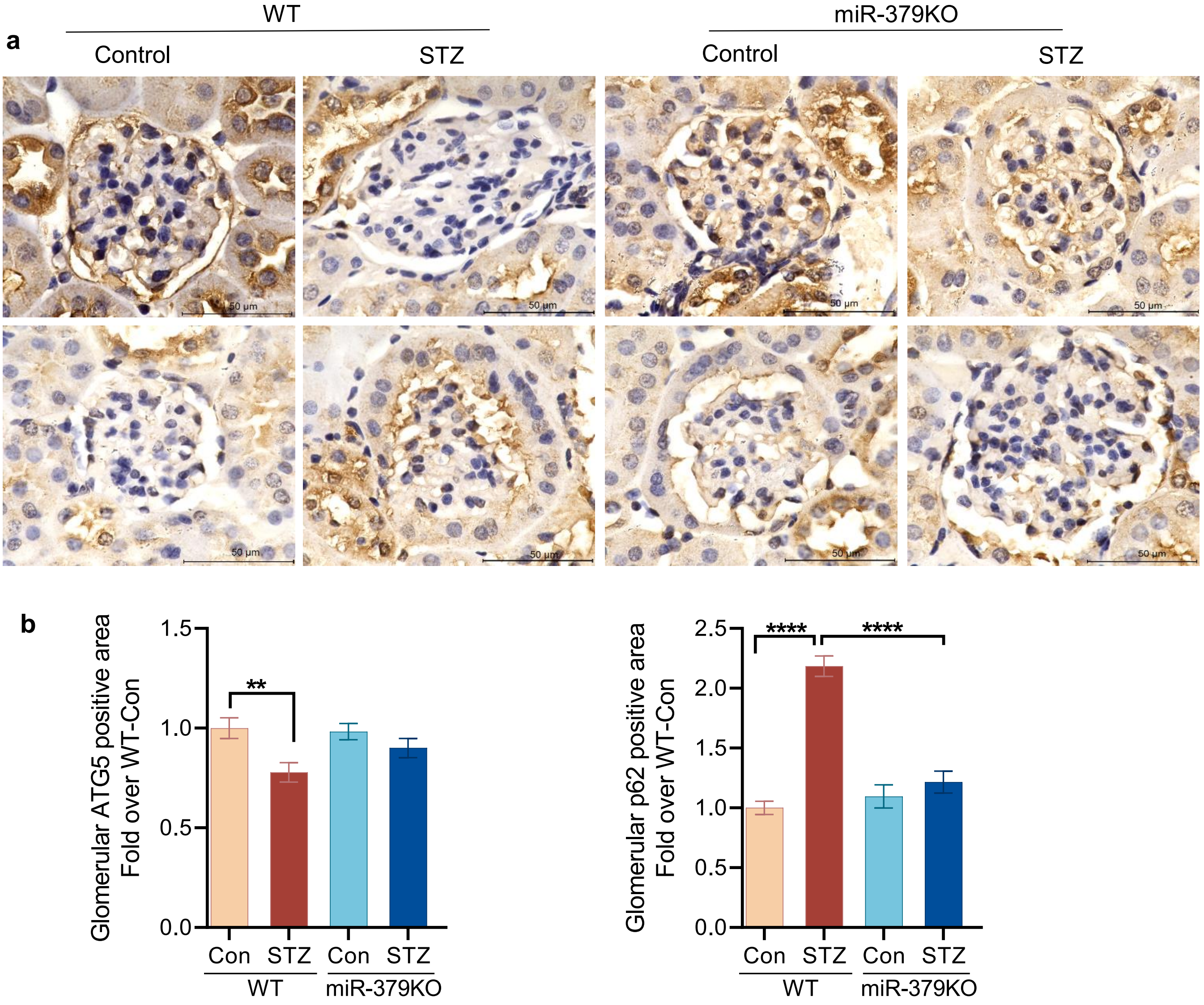

Supplementary Fig. 4 |Immunohistochemical staining and quantitative analysis of autophagy markers. a, Representative IHC images ATG5 and P62 protein in kidney cortex sections from WT and miR-379KO mice at 24 weeks after diabetes onset. **b**, quantitative data for ATG5 and P62. n=20 glomeruli/group. Scale bar, 50 μm. Bar graph results are expressed as fold over WT-Con. Statistical analyses were performed by One-way ANOVA with post-hoc Tukey test for multiple comparisons. *P < 0.05, ****P <0.0001. All data are presented as mean ± SEM.

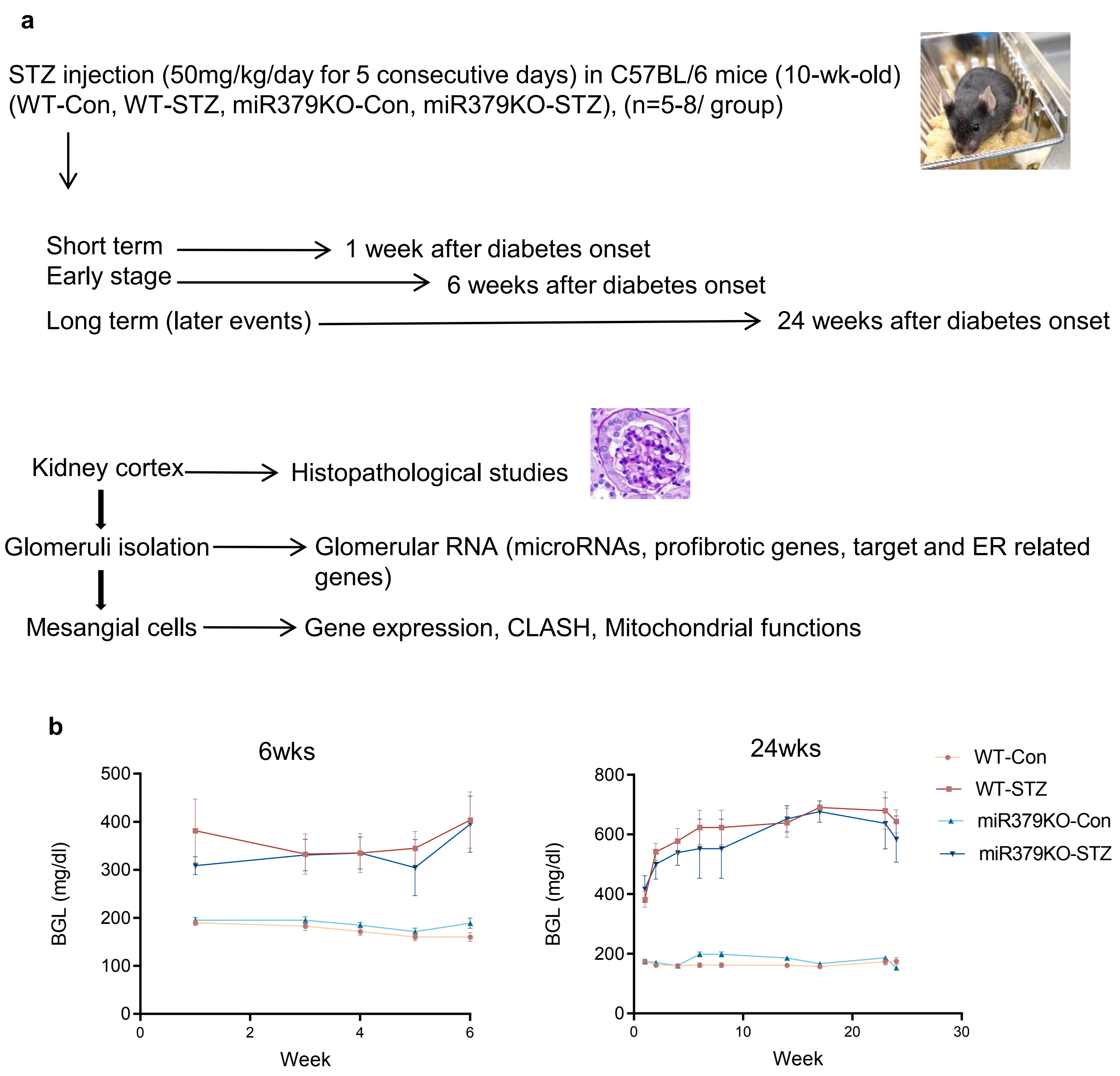

Supplementary Fig. 5 | Experimental design for theSTZ-injected T1D model.

a, Diabetes was induced with 50 mg/kg/day of streptozotocin (STZ) injected intraperitoneally on 5 consecutive days in 10-week-old male wild-type (WT-STZ) and miR-379KO (miR-379KO-STZ) C57BL/6 mice (n=5-8/group). Mice injected with diluent served as controls (WT-Con and miR-379KO-Con, n=5-8/group). Mice were sacrificed at 1, 6, or 24-week post-induction of diabetes. Sections from isolated kidney cortex were used for histopathological analysis. Isolated glomeruli were used for RNA expression by qPCR. Glomerular mesangial cells were used for RNA expression, CLASH analysis and mitochondrial functional assays. **b**, blood glucose was measured during 6 and 24 weeks experiment.

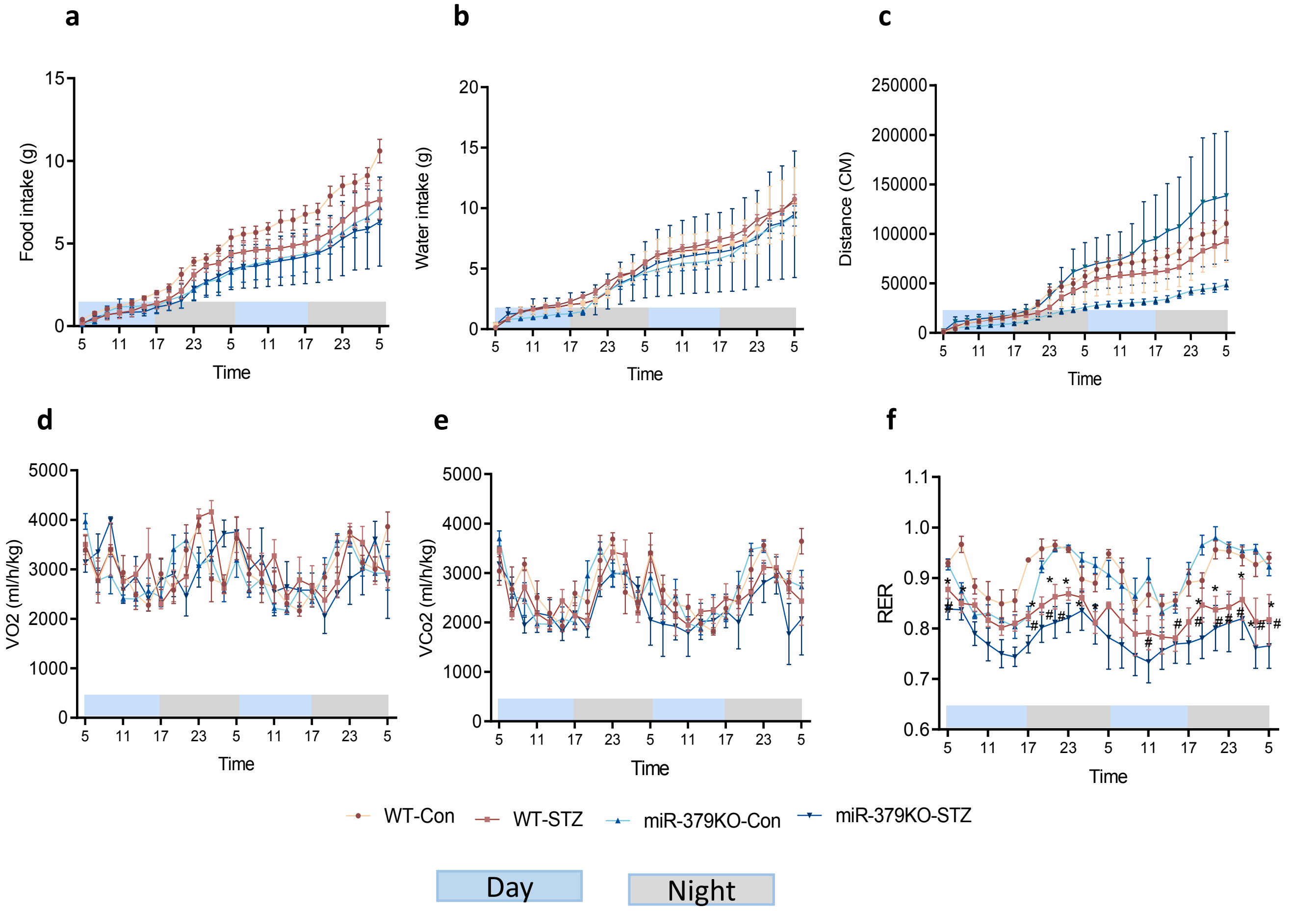

Supplementary Fig. 6 | Metabolic features measured using comprehensive metabolic cages over 3 days in WT and miR-379KO mice at 6 weeks after diabetes onset. Presented data from control and diabetic mice show average daily: **a**, food intake, **b,** water intake, **c**, movement (distance in cm), **d**, oxygen consumption (VO2), **e**, carbon dioxide production (VCO2) and **f**, respiratory exchange rate (RER). n=4/group for each individual experiment. Data points from individual mice were not plotted in the XY graphs to clearly show mean values and error bars. Statistical analyses were performed by Student's t-test. *$P < 0.05$ WT-STZ vs. WT-Con. $^{\#}P < 0.05$ miR-379KO-STZ vs. miR-379KO-Con. All data are presented as mean ± SEM.

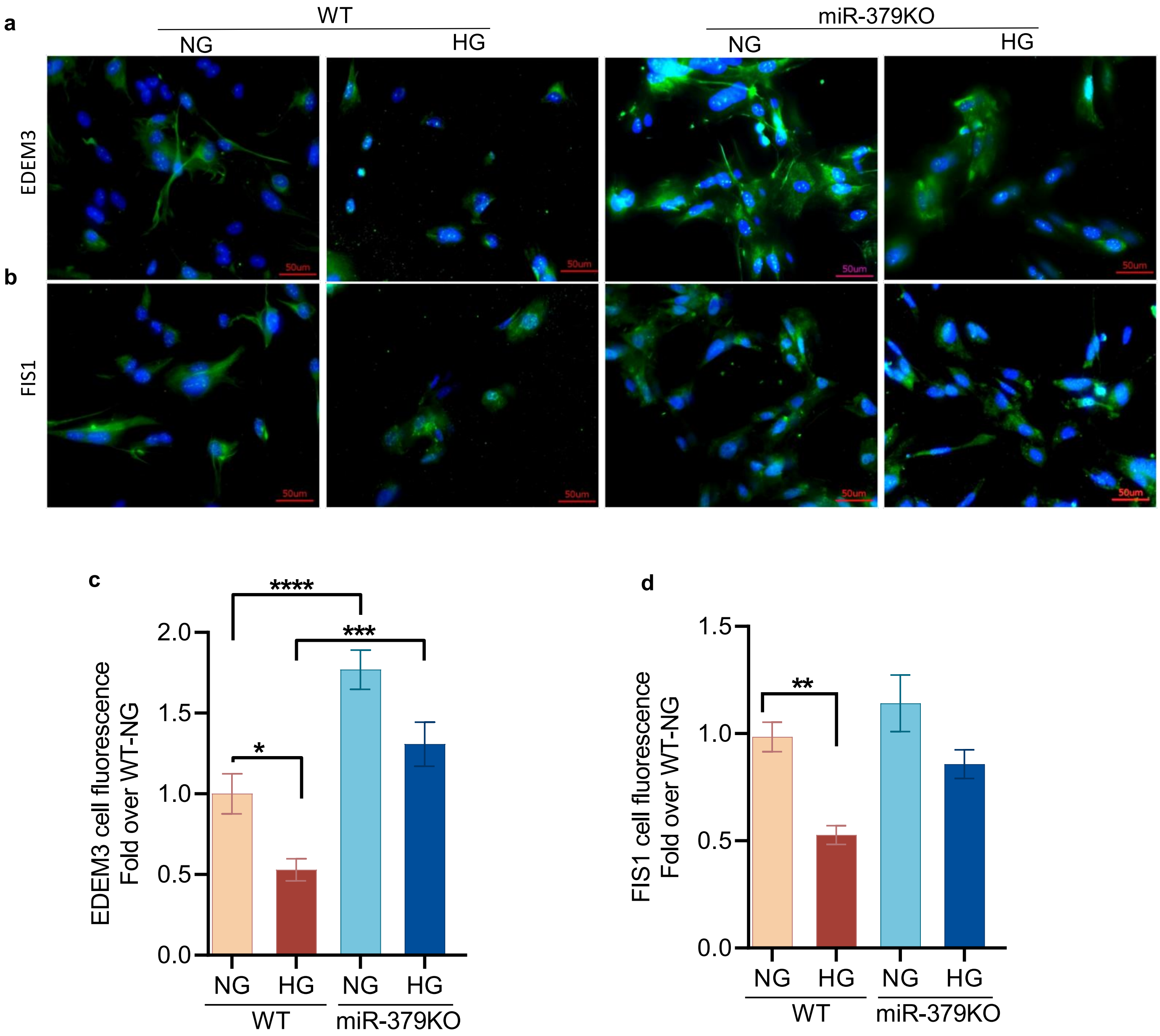

Supplementary Fig. 7| Immunofluorescence staining and quantitative analysis of miR-379 target proteins in MMC. Representative IF images **a**, EDEM3 and **b**,FIS1 protein in MMC (mesangial cells isolated from kidney cortex) from WT and miR-379KO mice. Decrease of EDEM3 and FIS1 (green) signals in WT MMC treated with HG (72 hours) compared to WT MMC NG, while no decrease of green signals in miR-379KO MMC in NG and even under HG conditions. Quantitative data for **c**, EDEM3 and **d**,FIS1. n= 15-20 cells/group. Scale bar, 50 μm. Bar graph results are expressed as fold over WT-NG. Statistical analyses were performed by One-way ANOVA with post-hoc Tukey test for multiple comparisons. $^{**}P < 0.01$, $^{***}P < 0.001$, $^{****}P < 0.0001$. All data are presented as mean ± SEM.

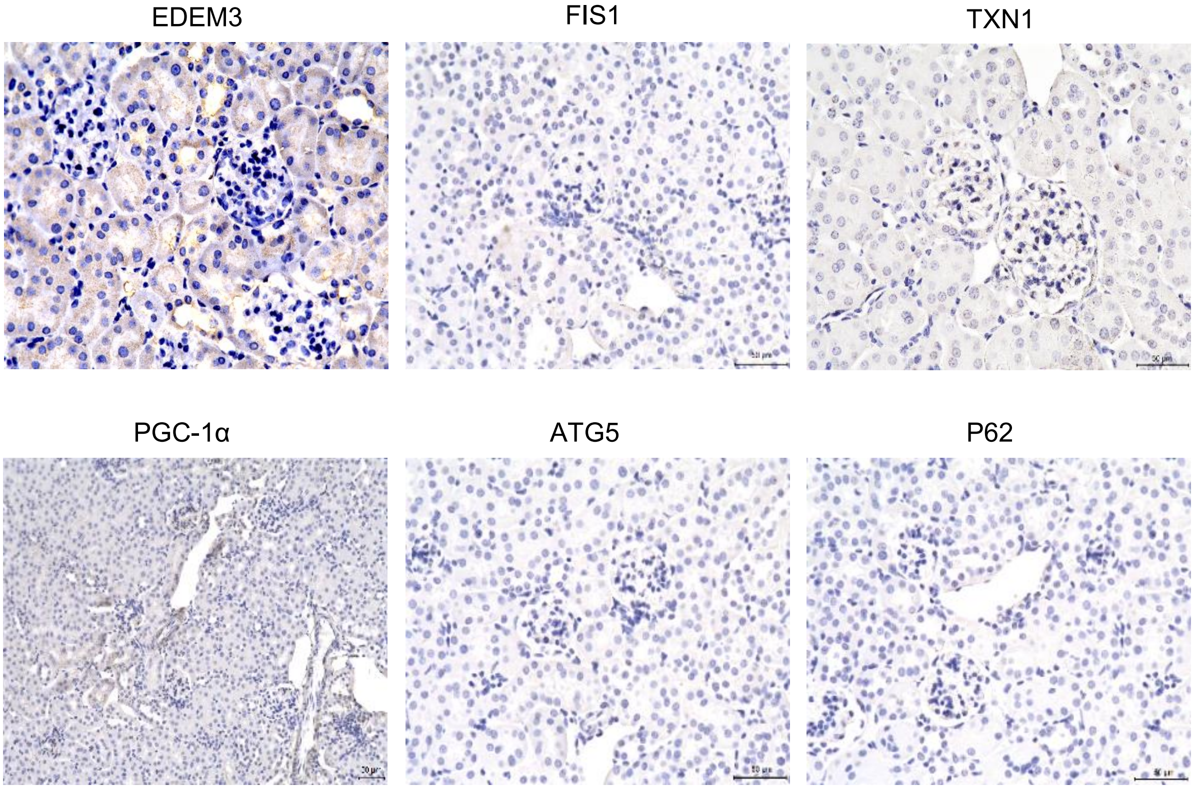

Supplementary Fig.8 | Controls for Immunohistochemical staining.

To rule out the background by the secondary antibody, control staining was performed without primary antibody using normal mouse kidney sections followed with incubation with Avidin: Biotinylated enzyme Complex (ABC) system and then DAB. No significant staining was detected without primary antibody for EDEM3, FIS1,TXN1,PGC-1α , ATG5, and P62. Scale bar, 50 μm.

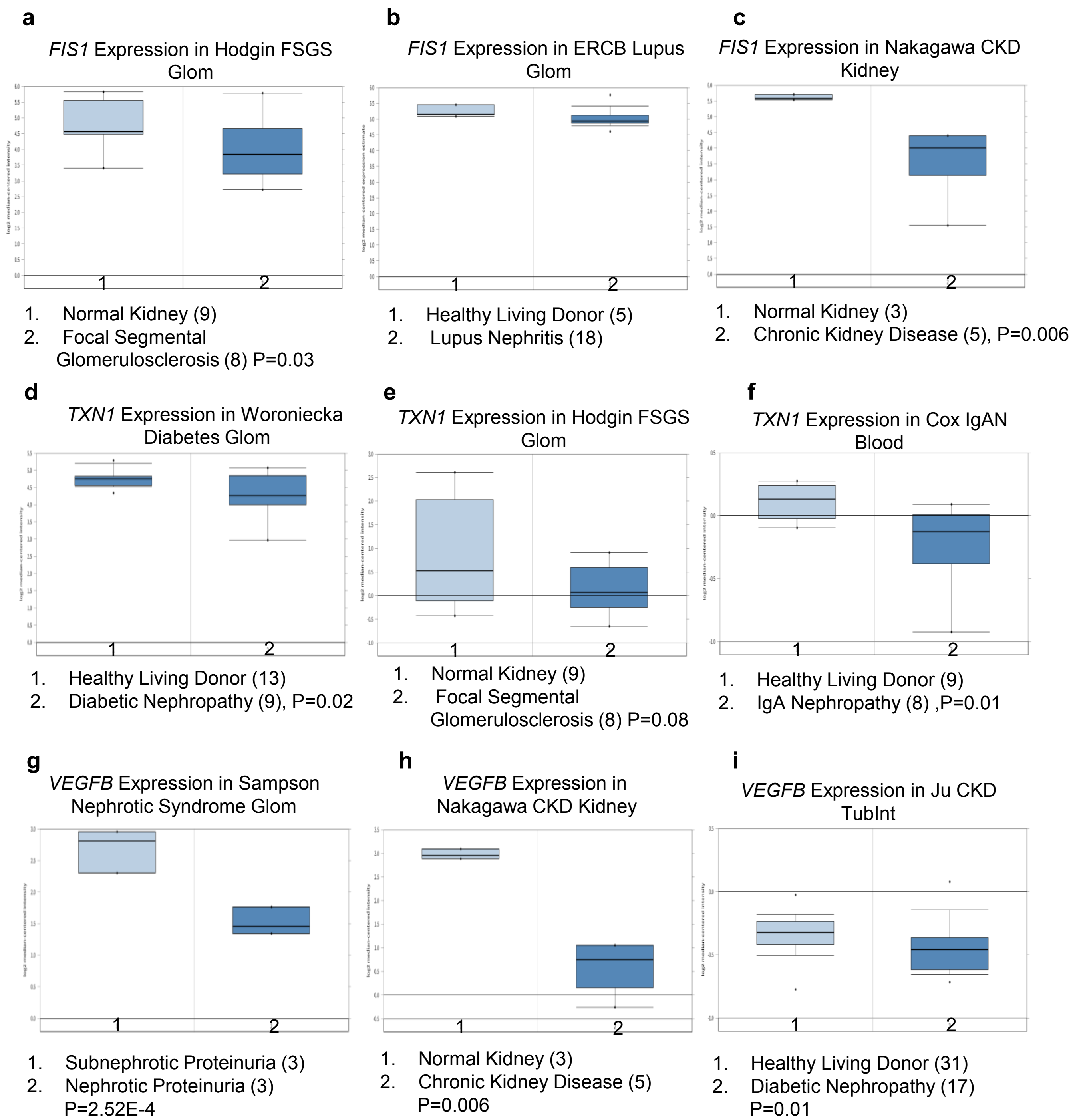

Supplementary Fig. 9 | Analysis of Nephroseq data for miR-379 target genes in human patients. *FIS1* expression in **a,** normal kidney (n=9) and focal segmental glomerulosclerosis (n=8), **b**, healthy living donors (n=5) and**,** lupus nephritis (n=18), and, **c,** normal kidney (n=3) and chronic kidney disease (n=5). *TXN1* expression in **d,** healthy living donors (n=13) and diabetic nephropathy (n=9), **e,** normal kidney (n=9) and focal segmental glomerulosclerosis (n=8), and **f,** healthy living donors (n=9) and IgA nephropathy (n=8) . *VEGFB* expression in **g,** subnephrotic proteinuria (n=3) and nephrotic proteinuria (n=3), **h,** normal kidney (n=3) and chronic kidney disease (n=5), and **I,** healthy living donors (n=31) and diabetic nephropathy (n=17).

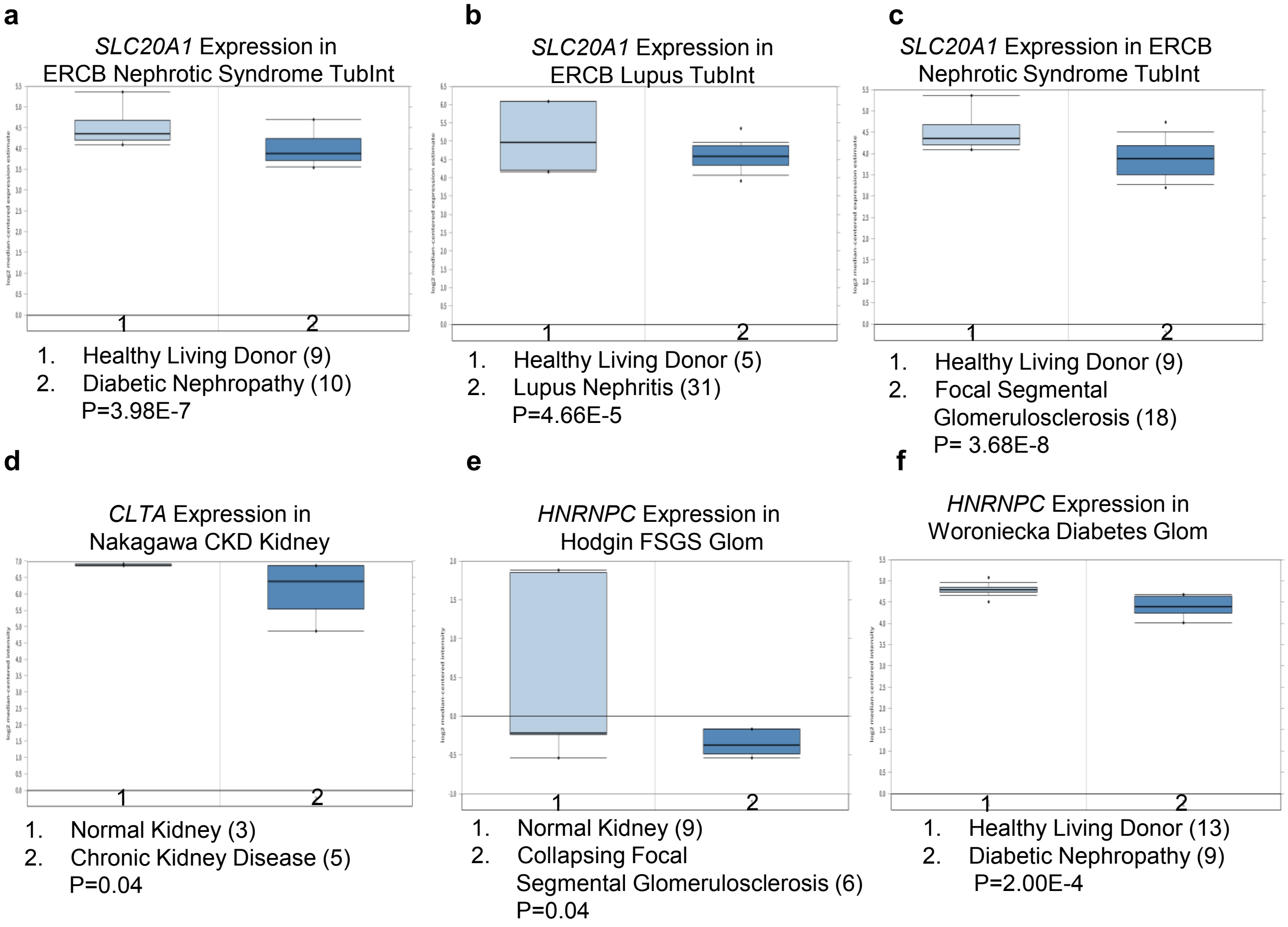

Supplementary. Fig. 10| Nephroseq data for miR-379 target genes in human patients. *SLC20A1* expression in , **a,** healthy living donors (n=9) and diabetic nephropathy (n=10) , **b**, healthy living donors (n=5) and lupus nephritis (n=31), and **c,** healthy living donors (n=9) and focal segmental glomerulosclerosis (n=18) . **d**, *CLTA* expression in normal kidney (n=3) and chronic kidney disease (n=5). *HNRNPC* expression in **e,** normal kidney (n=9) and collapsing focal segmental glomerulosclerosis (n=6) and, f**,** healthy living donors (n=13), and diabetic nephropathy (n=9).

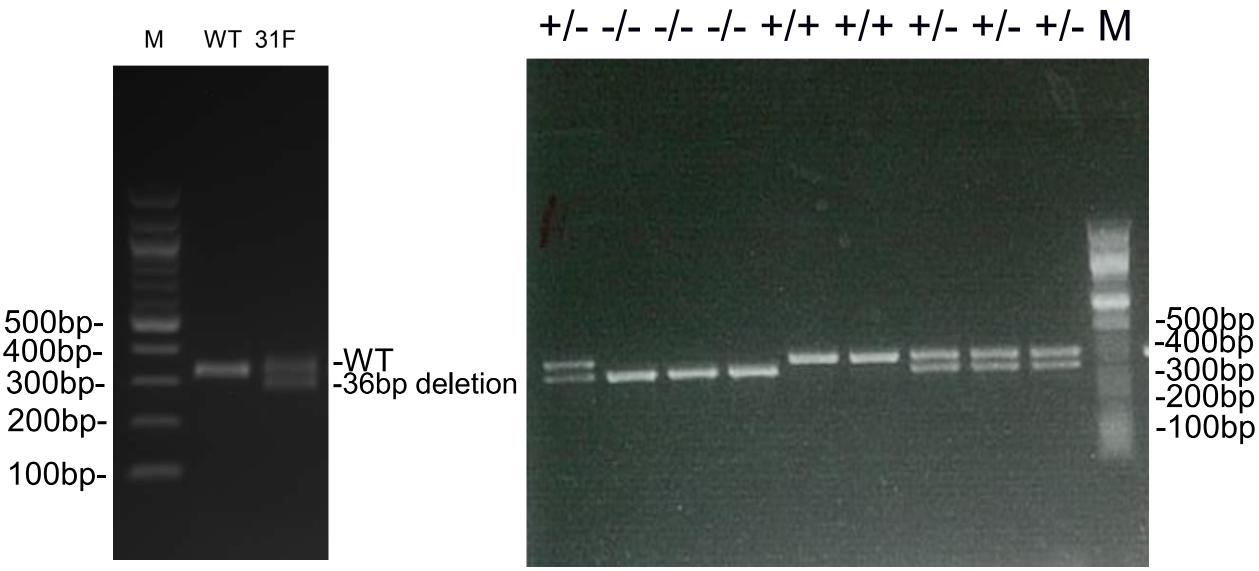

Supplementary. Fig. 11| Uncropped wider scans of gel pictures in Figure 1C.

Supplementary Table 1 | Summary of hybrid sequences fused with miR-379 identified by CLASH.

Read	Reads count	miRNA	miRNA beg	miRNA end	Gene	Target RNA beg	Target RNA end
ATTTCTGTATTTCCTTCTGCTCCTG TGTACCATGGTCCCCATACATTCT TGTAAATAAGGCTGCTATGAACAT AGTGGAGCA	1	mmu-miR-379-3p	26	38	*Slc7a11*	49	82
AACTTCATTAGAGACTGGTAGACT ATGGAACGTAGGGTAAGGAGTGC TCCTGCCCTCTAGTGGCTAGAGTT TAGGGTTGCAGTCTCTCCTGCACC CCTGTAGTCTACCT	6	mmu-miR-379-5p	20	34	*Fis1*	88	112
TGGTAGACTATGGAACGTAGAAGA AACACCACAGTACAATCGTCTACC ATTGGATGAAGTGGCTCCGGAGA TC	4	mmu-miR-379-5p	1	20	*Rprd2*	21	73

Partial List of hybrid sequences identified by CLASH.

Entire list of hybrid sequences is available as GEO # GSE14259.8

Actual DNA sequences identified by sequencing are shown in the Read column. The positions of beginning and end of miRNAs found in hybrid seq are shown as miRNA beg and end. The positions of beginning and end of target RNA found in the hybrid seq are shown as Target RNA beg and Target RNA end.

a **Supplementary Table 2 | Candidates of miR-379 targets**.

Candidate RNAs	Log2 (TAR/NT3UTR) WT-A_IP.	Log2 (TAR/NT3 UTR) WT-B_IP.	Log2 (TAR/NT3UTR) 379KO-A_IP.	Log2 (TAR/NT3UTR) 379KO-B_IP.	WT (Average of WT-A and WT-B)	KO (Average of 379KO-A and 379KO-B)	WT-KO	**Fold change (calculated from WT-KO)**
Clta	2.003924	1.701273	-0.12614	-0.14946	1.852598	-0.1378	1.990397	**3.973463**
Fis1	1.282478	1.208693	-0.21804	0.342257	1.245586	0.062106	1.183479	**2.271238**
Vegfb	2.276106	2.044893	0.902405	1.247112	2.1605	1.074759	1.085741	**2.122466**
Ap3s1	2.080585	2.450011	1.492658	1.315009	2.265298	1.403833	0.861465	**1.816882**
Hnrnpc	1.412723	1.726675	1.081704	1.04241	1.569699	1.062057	0.507642	**1.421725**
Txn1	3.580309	2.627671	2.869199	2.538936	3.10399	2.704067	0.399923	**1.319438**
Slc20a1	1.739989	1.648513	0.906599	2.008699	1.694251	1.457649	0.236602	**1.178214**

a, Ranking of RNA enrichment in AGO2 RISC complex in WT MMC based on fold changes in WT over KO. The local enrichment of each target region (TAR) relative to its own non-target 3'UTR region (NT3UTR) was calculated using log2 (TAR/NT3UTR) in each sample. Shown are candidate miR-379 targets identified by AGO2-IP RNA-seq and ranked by significant decrease of enrichment in AGO2-IP in miR-379KO MMC compared to WT MMC (bold column). WT, Average of WT-A and WT-B; KO, Average of 379KO-A and 379KO-B. A and B are replicate. WT-KO is WT minus KO. Fold change was calculated from the WT-KO. Wild type (WT), miR379KO (KO).

b

Candidate RNAs	Log2 (TAR/NT3UTR) WT-A_IP.	Log2 (TAR/NT3U TR) WT-B_IP.	Log2 (TAR/NT3UTR) 379KO-A_IP.	Log2 (TAR/NT3UTR) 379KO-B_IP.	**WT (Average of WT-A and WT-B)**	KO (Average of 379KO-A and 379KO-B)	WT-KO	Fold change (calculated from WT-KO)
Txn1	3.580309	2.627671	2.869199	2.538936	**3.10399**	2.704067	0.399923	1.319438
Ap3s1	2.080585	2.450011	1.492658	1.315009	**2.265298**	1.403833	0.861465	1.816882
Vegfb	2.276106	2.044893	0.902405	1.247112	**2.1605**	1.074759	1.085741	2.122466
Clta	2.003924	1.701273	-0.12614	-0.14946	**1.852598**	-0.1378	1.990397	3.973463
Slc20a1	1.739989	1.648513	0.906599	2.008699	**1.694251**	1.457649	0.236602	1.178214
Hnrnpc	1.412723	1.726675	1.081704	1.04241	**1.569699**	1.062057	0.507642	1.421725
Fis1	1.282478	1.208693	-0.21804	0.342257	**1.245586**	0.062106	1.183479	2.271238

b, Ranking of RNA enrichment in AGO2 RISC complex in WT MMC based on enrichment in WT. The local enrichment of each target region (TAR) relative to its own non-target 3'UTR region (NT3UTR) was calculated using log2 (TAR/NT3UTR) in each sample. Shown are candidate miR-379 targets identified by AGO2-IP RNA-seq and ranked by enrichment in AGO2-IP in WT MMC (bold column). WT, Average of WT-A and WT-B; KO, Average of 379KO-A and 379KO-B. A and B are replicate. WT-KO is WT minus KO. Fold change was calculated from the WT-KO. Wild type (WT), miR379KO (KO).

Supplementary Table 3

Oligonucleotide (DNA) sequences for potential target sites and guide RNAs at miR-379 region.

Target	Sense oligo	Antisense oligo
S1<br>CCTGAAGAGATGGTAGACTATGG	S1S<br>CACCgCCTGAAGAGATGGTAGACTA	S1AS<br>AAACTAGTCTACCATCTCTTCAGGc
S2<br>GATGGTAGACTATGGAACGTAGG	S2S<br>CACCgGATGGTAGACTATGGAACGT	S2AS<br>AAACACGTTCCATAGTCTACCATCc
S3<br>TGTTTTTGACCTATGTAACATGG	S3S<br>CACCgTGTTTTTGACCTATGTAACA	S3AS<br>AAACTGTTACATAGGTCAAAAACAc
AS1<br>CCTATGTAACATGGTCCACTAAC	AS1S<br>CACCgGTTAGTGGACCATGTTACAT	AS1AS<br>AAACATGTAACATGGTCCACTAACc
AS2<br>CCACTAACTCTCAGTATCCAATC	AS2S<br>CACCgGATTGGATACTGAGAGTTAG	AS2AS<br>AAACCTAACTCTCAGTATCCAATCc

Oligonucleotide (DNA) sequences for potential target sites and guide RNAs at the miR-379 region. Small letters g (sense) and c (antisense) show the positions of transcription start site.

Supplementary. Table 4

Primer sequences for qPCR of indicated targets used in the current study.

Target	Forward primer	Reverse primer
mSlc20a1	AGAAGCAGGGAGACAAAGC	GCCCAAAGTTCACATTCCAC
mAp3s1	GTATAAAAGTGCCAAACCTGCC	TGGTAAACTTAGATGACGTTCCC
mVegfb	GAACCTCATGTCTCACCTCAG	TCATAACAGAACCCAAATCCCG
mClta	CAGCAAACAGGCCAAAGATG	GTAGACGTAGTGTTTCCACAGG
mTxn1	ATGCCGACCTTCCAGTTTTAT	TGGTAGCTGGTTACACTTTTCAG
mFis1	ACCATCGCCTTCCCTTTTC	ATGACAGGGTTAAACGACAGG
mEdem3	TGAGTTCACTAGGAAATTCCAACA	CTGTGGTGATCATTTCAGCTCTA
mCol4a1	GCCTTCCGGGCTCCTCAG	CGGATTATCACCAGTGGGTCCG
mTgfb1	GGACTCTCCACCTGCAAGAC	GACTGGCGAGCCTTAGTTTG
mCtgf	AGTTTGTTATGGGTTAGGATTGTAG	CCAACACCAAAAAATAAAACCTCTA
mAp3s1	GTATAAAAGTGCCAAACCTGCC	TGGTAAACTTAGATGACGTTCCC
mHnrnpc	AGCTGGAGTTGAAGGATGATG	CTTTCATCTCCCACAAACGC
mHmgb1	GAAGACCTGAGAATGTATCCCC	AGCCAGCGTTCTTGTGATAG
mRab14	TCTACTCCATCTTTGCACTGTG	ACCCAGCCTTCTTCCTTTTC
mSnrpe	GGTGAAGTCTGTAGGATGTTGAG	AAGTTGAGTAAATGCAGGTTGC
mTcea1	ATGCTCTGCTTTGTAGACCG	TTCTGGGATTAAAGGTGTGGG
mAp3s1	GTATAAAAGTGCCAAACCTGCC	TGGTAAACTTAGATGACGTTCCC
mChop	GCACCTATATCTCATCCCCAG	TGCGTGTGACCTCTGTTG

Primer sequences for RT-qPCRs of indicated targets used in the current study
