# Supplementary material for: miR-379 deletion ameliorates features of diabetic kidney disease by enhancing adaptive mitophagy via FIS1
Source: Commun Biol. 2021 Jan 4;4:30. doi: 10.1038/s42003-020-01516-w (PMC7782535; doi:10.1038/s42003-020-01516-w)
Supplement: Supplementary file 2 — Description of Additional Supplementary Files [file 42003_2020_1516_MOESM2_ESM.pdf]

Description of Additional Supplementary Files

File name: Supplementary Data 1

Description: Source Data – Part 1

File name: Supplementary Data 2

Description: Source Data – Part 2
